# Circulating vitamin D concentration and risk of seven cancers: Mendelian randomisation study

**DOI:** 10.1136/bmj.j4761

**Published:** 2017-10-31

**Authors:** Vasiliki I Dimitrakopoulou, Konstantinos K Tsilidis, Philip C Haycock, Niki L Dimou, Kawthar Al-Dabhani, Richard M Martin, Sarah J Lewis, Marc J Gunter, Alison Mondul, Irene M Shui, Evropi Theodoratou, Katharina Nimptsch, Sara Lindström, Demetrius Albanes, Tilman Kühn, Timothy J Key, Ruth C Travis, Karani Santhanakrishnan Vimaleswaran, the GECCO Consortium, the PRACTICAL Consortium, the GAME-ON Network (CORECT, DRIVE, ELLIPSE, FOCI-OCAC, TRICL-ILCCO), Peter Kraft, Brandon L Pierce, Joellen M Schildkraut

**Affiliations:** 1 Department of Hygiene and Epidemiology, School of Medicine, University of Ioannina, Ioannina, Greece; 2 School of Mathematics and Statistics, University College Dublin, Dublin, Ireland; 3 Department of Epidemiology and Biostatistics, School of Public Health, Imperial College London, London, UK; 4 School of Social and Community Medicine, University of Bristol, Bristol, UK; 5 MRC Integrative Epidemiology Unit, University of Bristol, Bristol, UK; 6 National Institute for Health Research (NIHR) Bristol Nutritional Biomedical Research Unit, University Hospitals Bristol NHS Foundation Trust and the University of Bristol, Bristol, UK; 7 International Agency for Research on Cancer, Lyon, France; 8 Department of Epidemiology, University of Michigan School of Public Health, Ann Arbor, MI, USA; 9 Department of Epidemiology, Harvard School of Public Health, Boston, MA, USA; 10 Centre of Global Health Research, Usher Institute for Population Health Sciences and Informatics, University of Edinburg, Edinburgh, UK; 11 Molecular Epidemiology Research Group, Max Delbrück Centre for Molecular Medicine (MDC), Berlin, Germany; 12 Department of Epidemiology, University of Washington, Seattle, WA, USA; 13 Division of Cancer Epidemiology and Genetics, National Cancer Institute, Bethesda, MD, USA; 14 Division of Cancer Epidemiology, German Cancer Research Centre (DKFZ), Heidelberg, Germany; 15 Cancer Epidemiology Unit, Nuffield Department of Population Health, University of Oxford, Oxford, UK; 16 Department of Food and Nutritional Sciences, Hugh Sinclair Unit of Human Nutrition and Institute for Cardiovascular and Metabolic Research (ICMR), University of Reading, Reading, UK; 17 Program in Genetic Epidemiology and Statistical Genetics, Department of Epidemiology, Harvard School of Public Health, Boston, MA, USA; 18 Department of Public Health Sciences, University of Chicago, Chicago, IL, USA; 19 Comprehensive Cancer Center, University of Chicago, Chicago, IL, USA; 20 Department of Human Genetics, University of Chicago, Chicago, IL, USA; 21 Department of Public Health Sciences, University of Virginia, Charlottesville, VA, USA

## Abstract

**Objective** To determine if circulating concentrations of vitamin D are causally associated with risk of cancer.

**Design** Mendelian randomisation study.

**Setting** Large genetic epidemiology networks (the Genetic Associations and Mechanisms in Oncology (GAME-ON), the Genetic and Epidemiology of Colorectal Cancer Consortium (GECCO), and the Prostate Cancer Association Group to Investigate Cancer Associated Alterations in the Genome (PRACTICAL) consortiums, and the MR-Base platform).

**Participants** 70 563 cases of cancer (22 898 prostate cancer, 15 748 breast cancer, 12 537 lung cancer, 11 488 colorectal cancer, 4369 ovarian cancer, 1896 pancreatic cancer, and 1627 neuroblastoma) and 84 418 controls.

**Exposures** Four single nucleotide polymorphisms (rs2282679, rs10741657, rs12785878 and rs6013897) associated with vitamin D were used to define a multi-polymorphism score for circulating 25-hydroxyvitamin D (25(OH)D) concentrations.

**Main outcomes measures** The primary outcomes were the risk of incident colorectal, breast, prostate, ovarian, lung, and pancreatic cancer and neuroblastoma, which was evaluated with an inverse variance weighted average of the associations with specific polymorphisms and a likelihood based approach. Secondary outcomes based on cancer subtypes by sex, anatomic location, stage, and histology were also examined.

**Results** There was little evidence that the multi-polymorphism score of 25(OH)D was associated with risk of any of the seven cancers or their subtypes. Specifically, the odds ratios per 25 nmol/L increase in genetically determined 25(OH)D concentrations were 0.92 (95% confidence interval 0.76 to 1.10) for colorectal cancer, 1.05 (0.89 to 1.24) for breast cancer, 0.89 (0.77 to 1.02) for prostate cancer, and 1.03 (0.87 to 1.23) for lung cancer. The results were consistent with the two different analytical approaches, and the study was powered to detect relative effect sizes of moderate magnitude (for example, 1.20-1.50 per 25 nmol/L decrease in 25(OH)D for most primary cancer outcomes. The Mendelian randomisation assumptions did not seem to be violated.

**Conclusions** There is little evidence for a linear causal association between circulating vitamin D concentration and risk of various types of cancer, though the existence of causal clinically relevant effects of low magnitude cannot be ruled out. These results, in combination with previous literature, provide evidence that population-wide screening for vitamin D deficiency and subsequent widespread vitamin D supplementation should not currently be recommended as a strategy for primary cancer prevention.

## Introduction

Evidence from in vitro and animal model studies supports an anti-neoplastic role of vitamin D.^1^ Vitamin D functions by activating the nuclear vitamin D receptor, which is ubiquitously expressed and regulates the growth, differentiation, and apoptosis of normal and tumour cells.^1^

Epidemiological studies of circulating vitamin D concentrations and risk of various cancers have produced inconsistent results. Meta-analyses of observational studies have suggested that higher concentrations of 25-hydroxyvitamin D (25(OH)D), the primary circulating form, is associated with a lower risk of colorectal cancer.^2^ Epidemiological evidence for breast and prostate cancer is inconclusive, while data for other cancers are limited.^3^ ^4^ ^5^ Previous observational associations between circulating 25(OH)D and cancer are limited by relatively small study specific sample sizes (for example, 3000-5000 cases in meta-analyses of breast, prostate, and colorectal cancer) and by several potential methodological issues. Specifically, reverse causation could exist if 25(OH)D is measured at or close to cancer diagnosis, residual confounding might be present because of inadequate control for common causes of cancer, and errors in measurement of exposure to 25(OH)D could result from single measurements.

Definitive data from randomised controlled trials are lacking as few adequately powered trials have examined vitamin D supplementation and risk of cancer. The Women’s Health Initiative,^6^ a randomised placebo controlled trial of 400 IU of vitamin D plus 1000 mg of calcium per day in 36 282 postmenopausal women, failed to support a protective role of vitamin D over a period of seven years for colorectal cancer (n=332 cases), breast cancer (n=1074 cases), or total cancer (n=2639 cases). The dose of vitamin D, however, was probably inadequate and the follow-up was too short to yield a substantial contrast. A meta-analysis of four vitamin D supplementation trials found no association with total cancer incidence.^7^ Another meta-analysis of 18 trials found a decrease in total cancer mortality, but the possibility of type I error and attrition bias was reported as few participants were examined and there was substantial dropout.^8^ A previous Mendelian randomisation study reported that genetically low 25(OH)D concentrations were associated with increased cancer mortality, but this study included only about 2800 deaths from cancer and could not perform analyses by cancer site.^9^

To overcome limitations of conventional observational research and randomised trials and shed light on whether vitamin D status is a cause of disease or just a correlate marker of overall health, we used a Mendelian randomisation approach and estimated associations between single nucleotide polymorphisms associated with vitamin D and risk of colorectal, breast, prostate, ovarian, lung, and pancreatic cancer and neuroblastoma using summary data from the Genetic Associations and Mechanisms in Oncology (GAME-ON), the Genetic and Epidemiology of Colorectal Cancer Consortium (GECCO), and the Prostate Cancer Association Group to Investigate Cancer Associated Alterations in the Genome (PRACTICAL) consortiums, and the MR-Base platform. Mendelian randomisation aims to improve causal inference by assessing risk associations of the genetically determined component of environmental exposures and biomarkers.^10^ ^11^

## Methods

### Data for genetic epidemiology of cancer

We retrieved summary data for the association between single nucleotide polymorphisms associated with vitamin D and cancer from three large genetic epidemiology networks. The GAME-ON initiative is a network of five cancer specific consortiums: CORECT (ColoRectal Transdisciplinary Study); DRIVE (Discovery, Biology, and Risk of Inherited Variants in Breast Cancer); ELLIPSE (Elucidating Loci Involved in Prostate Cancer Susceptibility); FOCI-OCAC (Follow-up of Ovarian Cancer Genetic Association and Interaction Studies of the Ovarian Cancer Association Consortium); and TRICL-ILCCO (Transdisciplinary Research in Cancer of the Lung of the International Lung Cancer Consortium). Larger scale summary data on the genetic epidemiology of colorectal and prostate cancer were retrieved from the GECCO and PRACTICAL consortiums. Further details on these networks can be found elsewhere.^12^ ^13^ ^14^ Data for the genetic epidemiology of pancreatic cancer and neuroblastoma were retrieved from PanScan1 (Pancreatic Cancer Cohort Consortium Genome-Wide Association Study) and from a genome-wide association study of neuroblastoma through the MR-Base platform.^15^ ^16^ ^17^ ^18^

Results from individual genome-wide association studies for each cancer type were combined by using standard fixed effects meta-analysis methods. We used Illumina or Affymetrix arrays for genotyping and either MACH^19^ or IMPUTE^20^ for imputation with the 1000 Genomes reference panel. We incorporated principal components as covariates in the single nucleotide polymorphism and cancer logistic regression models to adjust for population stratification. Further information regarding the statistical analysis, imputation, and quality control steps in the genome-wide association studies have been previously reported.^16^ ^17^ ^21^ ^22^ ^23^ ^24^

### Data for genetic epidemiology of circulating 25(OH)D concentrations

We conducted a search of published genome-wide association studies in PubMed and the relevant catalogue and identified four single nucleotide polymorphisms as robustly associated at P<5×10^−8^ with circulating 25(OH)D concentration in two genome-wide association studies.^25^ ^26^ These were rs2282679 in the group specific component (GC) on chromosome 4p12 that encodes the vitamin D binding protein; rs10741657 in CYP2R1 on chromosome 11p15 that is involved in the hydroxylation of vitamin D3 to 25(OH)D; rs12785878 located near DHCR7 on chromosome 11q12 that catalyses the conversion of 7-dehydrocholesterol to cholesterol; and rs6013897 near CYP24A1 on chromosome 20q13 that encodes an enzyme that initiates the degradation of 1,25(OH)_2_D. All four single nucleotide polymorphisms were identified among individuals of European ancestry. Each explained about 1% of the 25(OH)D variability, and up to 5% for combinations of the four polymorphisms.^25^ ^26^ ^27^ More precise estimates of the associations between the polymorphisms with circulating 25(OH)D were obtained from a recent large Mendelian randomisation study.^28^ That study analysed the association between the aforementioned polymorphisms with circulating 25(OH)D concentrations as per unit change (nmol/L) in the natural (untransformed) scale.

### Statistical analysis

We conducted Mendelian randomisation analyses to test the potential causal associations between circulating 25(OH)D and the risk of seven cancers (colorectal, breast, prostate, ovarian, lung, pancreatic, and neuroblastoma) using summary data from GAME-ON, GECCO, PRACTICAL, MR-Base, and genome-wide association studies of 25(OH)D concentration. We also performed analyses for cancer subtypes: colorectal cancer in men and women, colon cancer, rectal cancer, proximal colon cancer, distal colon cancer, oestrogen receptor negative breast cancer; aggressive prostate cancer^24^ ^29^; clear cell, endometrioid, and serous ovarian cancer; and adenocarcinoma and squamous cell carcinoma of lung. We formulated a weighted multi-polymorphism score, which has been previously shown to be linearly associated with circulating 25(OH)D concentration.^28^ We used two Mendelian randomisation methods using summary genetic data: an inverse variance weighted average of associations for specific polymorphisms and a likelihood based method.^30^ ^31^ More information on these methods is provided in appendix 1.

For the Mendelian randomisation analyses to have a valid interpretation, it is necessary that the following three instrumental variable assumptions hold^32^ ^33^: the genetic markers are strongly associated with circulating vitamin D concentration; the markers affect cancer only through their effect on circulating vitamin D; and markers are independent of any confounders of the association between circulating vitamin D and cancer. To assess potential violation of these assumptions we performed several statistical tests (MR-Egger,^34^ weighted median approach,^35^ and over-identification tests^36^) and sensitivity analyses (Mendelian randomisation analyses with two separate allelic scores: a vitamin D synthesis allele score (rs10741657 and rs12785878) and a metabolism allele score (rs2282679 and rs6013897)), more information about which is provided in appendix 1.

## Results

### Descriptives and statistical power

Table 1 lists the samples sizes used in the current study for each cancer type. The number of cancer cases ranged from 1627 for neuroblastoma to 22 898 for prostate cancer. Our Mendelian randomisation analyses had 80% power, assuming that 3% of the 25(OH)D variance was explained by the four single nucleotide polymorphisms, to detect effect sizes of moderate magnitude, ranging from odds ratios of 0.58 per SD (for instance, 25 nmol/L or 10 ng/mL) increase in circulating 25(OH)D concentration for neuroblastoma to 0.86 for prostate cancer (table 1), which are comparable with effect sizes that have been observed in observational studies relating circulating 25(OH)D concentration to risk of cancer. Similar minimum detectable effect sizes were estimated for cancer subtypes, except for clear cell and endometrioid ovarian carcinomas, for which there was adequate power to detect only large effects (for example, odds ratios 0.19-0.43). The power was larger if we assumed that 5% of the 25(OH)D variance was explained by the single nucleotide polymorphisms (table 1). Table 2 shows information on the associations of rs2282679, rs10741657, rs12785878 and rs6013897 with 25(OH)D concentration.

**Table 1 tbl1:** Number of cancer cases and controls and statistical power in Mendelian randomisation study of circulating vitamin D concentration and risk of seven cancers

Cancer type	Study	Cases	Controls	Minimum detectable OR* (*R^2^*=0.03)	Minimum detectable OR* (*R^2^*=0.05)	OR (95% CI) in published meta-analyses†
**Colorectal**						
All	GAME-ON	5100	4831	0.72/1.39	0.78/1.28	0.74 (0.63 to 0.89)^37^
All	GECCO	11 488	11 679	0.81/1.23	0.85/1.18	
All (women)	GECCO	6132	6380	0.75/1.33	0.80/1.25	NR
All (men)	GECCO	5356	5297	0.73/1.37	0.78/1.28	NR
Colon	GECCO	7678	11 679	0.78/1.28	0.83/1.20	NR
Rectal	GECCO	2783	11 679	0.68/1.47	0.75/1.33	NR
Distal colon	GECCO	3354	11 679	0.70/1.43	0.77/1.30	NR
Proximal colon	GECCO	4185	11 679	0.73/1.37	0.79/1.27	NR
**Breast**						
All	DRIVE	15 748	18 084	0.84/1.19	0.87/1.15	0.89 (0.81 to 0.98)^38^
ER−	DRIVE	4939	13 128	0.75/1.29	0.80/1.22	NR
**Prostate**						
All	PRACTICAL	22 898	23 054	0.86/1.16	0.89/1.12	1.04 (0.99 to 1.10)^39^
All	GAME-ON	14 159	12 712	0.82/1.22	0.86/1.17	
Aggressive	GAME-ON	4445	12 724	0.74/1.30	0.79/1.23	0.98 (0.84 to 1.15)^39^
**Ovarian**						
All	FOCI	4369	9123	0.73/1.33	0.79/1.25	0.91 (0.79 to 1.04)^40^
Clear-cell	FOCI	356	9123	0.19/1.86	0.36/1.67	NR
Endometrioid	FOCI	715	9123	0.43/1.62	0.55/1.48	NR
Serous	FOCI	2556	9123	0.67/1.39	0.74/1.30	NR
**Lung**						
All	TRICL-ILCCO	12 537	17 285	0.82/1.20	0.86/1.16	0.98 (0.96 to 0.99)^41^
Adenocarcinoma	TRICL-ILCCO	3804	16 289	0.73/1.30	0.78/1.23	NR
Squamous	TRICL-ILCCO	3546	16 434	0.72/1.31	0.78/1.24	NR
**Pancreatic**						
All	PanScan1‡	1896	1939	0.59/1.67	0.67/1.49	NR
**Neuroblastoma**						
All	Capasso, et al^17^‡	1627	3254	0.58/1.57	0.66/1.43	NR

NR=not reported; ER−=oestrogen receptor negative.

*Minimum detectable odds ratio per 1 SD increase/decrease in 25(OH)D concentration; assume 80% power, 5% alpha level, and that 3% or 5% of 25(OH)D variance is explained by four single nucleotide polymorphisms (rs2282679, rs10741657, rs12785878, rs6013897) used in this paper. 1 SD in 25(OH)D corresponds to about 25 nmol/L (10 ng/mL).

†Summary random effects odds ratio and 95% confidence intervals for association of continuous 25(OH)D concentration (per 25 nmol/L) and risk of cancer in most recent published meta-analysis that reported dose-response summary result.

‡Obtained through MR-base platform.

**Table 2 tbl2:** Characteristics of genetic variants associated with 25(OH)D concentration in published genome-wide association studies*

Single nucleotide polymorphism	Chromosome	Locus	Risk allele	β estimate†	P value
rs2282679	4	GC	G	−4.67	<3.4×10^−302^
rs10741657	11	CYP2R1	G	−1.72	6.5×10^−81^
rs12785878	11	DHCR7/NADSYN1	G	−2.11	6.4×10^−129^
rs6013897	20	CYP24A1	A	−0.98	3.4×10^−17^

*Source: Vimaleswaran, et al, 2013.^28^

†Reported per unit change in nmol/L in natural scale per effect allele.

### Association between individual single nucleotide polymorphisms and cancer

Appendix 2 shows the association between each single nucleotide polymorphism related to vitamin D and risk of colorectal, breast, prostate, ovarian, lung, and pancreatic cancer and neuroblastoma and their subtypes with data from GAME-ON, GECCO, PRACTICAL, and the MR-Base platform. None of the four polymorphisms was significantly associated with any cancer risk, except for rs6013897 with prostate cancer in the GAME-ON data (odds ratio per effect allele 1.06, 95% confidence interval 1.01 to 1.10; P=0.02), but this association was not observed in the larger PRACTICAL data (1.00, 0.97 to 1.04; P=0.81). The rs6013897 polymorphism was also associated with risk of colon cancer in the GECCO data (0.94, 0.89 to 0.99; P=0.03).

### Mendelian randomisation estimates for multi-polymorphism scores

Based on Mendelian randomisation analyses with either the inverse variance weighted method or the likelihood based method, we found little evidence that the multi-polymorphism scores for continuous 25(OH)D concentration were associated with risk of colorectal, breast, prostate, ovarian, lung, or pancreatic cancer and neuroblastoma or their subtypes (table 3). Figures 1-6[Fig f1 f2 f3 f4 f5 f6] show scatter plots of associations between vitamin D polymorphism and risk of various types of cancer. Plots are overlaid by Mendelian randomisation estimate (slope of solid line) and its 95% confidence interval (dotted lines) of multi-polymorphism score of continuous circulating 25(OH)D on risk of the seven cancers and their subtypes. We found a marginally significant association for total prostate cancer, for which a genetically determined 25 nmol/L increase in 25(OH)D concentration yielded an odds ratio of 0.89 (95% confidence interval 0.77 to 1.02; P=0.08; fig 3). 

**Table 3 tbl3:** Mendelian randomisation estimates between multi-single nucleotide polymorphism risk scores of continuous 25(OH)D and risk of cancer calculated with inverse variance weighted method and likelihood method

Cancer type	Study	OR* (95% CI); P value	
		Inverse variance weighted	Likelihood
**Colorectal**			
All	GAME-ON	1.04 (0.78 to 1.38); 0.81	1.04 (0.78 to 1.38); 0.81
All	GECCO	0.92 (0.76 to 1.10); 0.36	0.92 (0.76 to 1.10); 0.36
All (women)	GECCO	0.92 (0.71 to 1.18); 0.52	0.92 (0.71 to 1.18); 0.52
All (men)	GECCO	0.91 (0.70 to 1.20); 0.52	0.91 (0.70 to 1.20); 0.52
Colon	GECCO	0.90 (0.73 to 1.11); 0.33	0.90 (0.73 to 1.11); 0.33
Rectal	GECCO	0.93 (0.68 to 1.26); 0.64	0.93 (0.68 to 1.26); 0.64
Distal colon	GECCO	0.97 (0.73 to 1.28); 0.83	0.97 (0.73 to 1.28); 0.83
Proximal colon	GECCO	0.83 (0.64 to 1.07); 0.14	0.82 (0.64 to 1.07); 0.14
**Breast**			
All	DRIVE	1.05 (0.89 to 1.24); 0.59	1.05 (0.89 to 1.24); 0.59
ER−	DRIVE	1.15 (0.88 to 1.50); 0.30	1.15 (0.88 to 1.50); 0.30
**Prostate**			
All	PRACTICAL	0.89 (0.77 to 1.02); 0.08	0.89 (0.77 to 1.02); 0.08
All	GAME-ON	1.08 (0.88 to 1.33); 0.47	1.08 (0.88 to 1.33); 0.46
Aggressive	GAME-ON	1.14 (0.85 to 1.54); 0.38	1.15 (0.85 to 1.54); 0.38
**Ovarian**			
All	FOCI	1.12 (0.86 to 1.47); 0.40	1.12 (0.86 to 1.47); 0.40
Clear-cell	FOCI	0.99 (0.46 to 2.11); 0.98	0.99 (0.46 to 2.11); 0.98
Endometrioid	FOCI	0.83 (0.48 to 1.43); 0.51	0.83 (0.48 to 1.43); 0.51
Serous	FOCI	1.26 (0.91 to 1.76); 0.17	1.26 (0.91 to 1.76); 0.17
**Lung**			
All	TRICL-ILCCO	1.03 (0.87 to 1.23); 0.72	1.03 (0.87 to 1.23); 0.72
Adenocarcinoma	TRICL-ILCCO	1.03 (0.79 to 1.35); 0.84	1.03 (0.79 to 1.35); 0.84
Squamous	TRICL-ILCCO	0.95 (0.72 to 1.25); 0.74	0.95 (0.72 to 1.25); 0.74
**Pancreatic**			
All	PanScan1†	1.36 (0.81 to 2.27); 0.25	1.36 (0.80 to 2.27); 0.25
**Neuroblastoma**			
All	Capasso, et al^17^†	0.76 (0.47 to 1.21); 0.24	0.76 (0.47 to 1.21); 0.24

ER−=oestrogen receptor negative.

*Represents increase/decrease of risk per 25 nmol/L increase in nmol/L in natural scale of 25(OH)D. All four single nucleotide polymorphisms were used for all cancers, except for pancreatic cancer and neuroblastoma, for which only two polymorphisms (rs10741657, rs2282679) were available.

†Obtained through MR-base platform.

**Figure f1:**
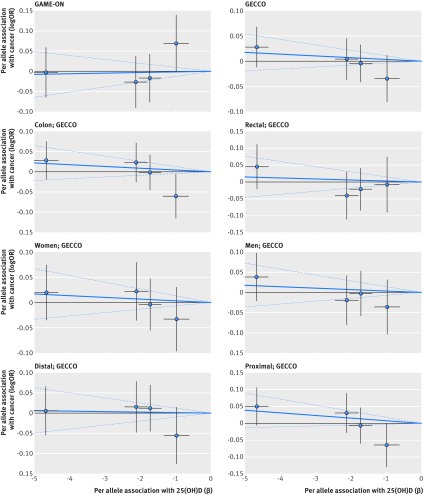
**Fig 1** Association between single nucleotide polymorphisms associated with vitamin D and risk of colorectal cancer and circulating 25(OH)D concentration. Per allele associations with risk plotted against per allele associations with continuous circulating 25(OH)D concentration (vertical and horizontal black lines around points show 95% confidence interval for each polymorphism)

**Figure f2:**
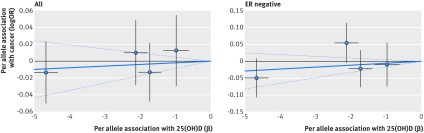
**Fig 2** Association between single nucleotide polymorphisms associated with vitamin D and risk of breast cancer and circulating 25(OH)D concentration. Per allele associations with risk plotted against per allele associations with continuous circulating 25(OH)D concentration (vertical and horizontal black lines around points show 95% confidence interval for each polymorphism)

**Figure f3:**
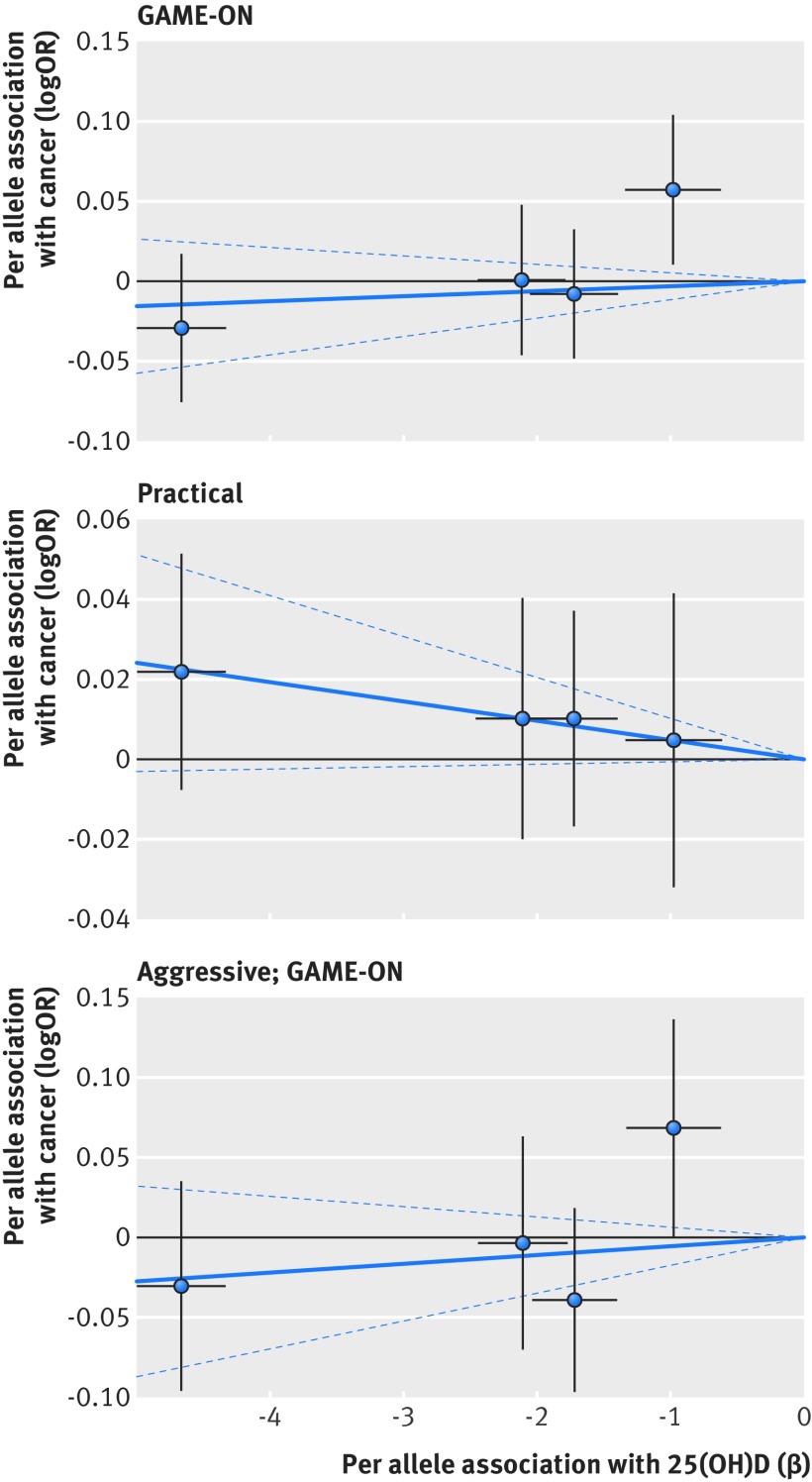
**Fig 3** Association between single nucleotide polymorphisms associated with vitamin D and risk of prostate cancer and circulating 25(OH)D concentration. Per allele associations with risk plotted against per allele associations with continuous circulating 25(OH)D concentration (vertical and horizontal black lines around points show 95% confidence interval for each polymorphism)

**Figure f4:**
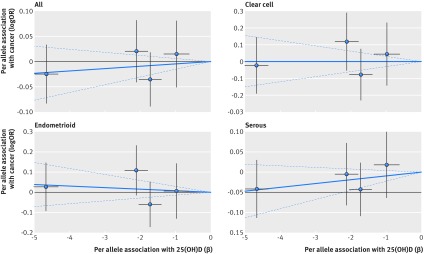
**Fig 4** Association between single nucleotide polymorphisms associated with vitamin D and risk of ovarian cancer and circulating 25(OH)D concentration. Per allele associations with risk plotted against per allele associations with continuous circulating 25(OH)D concentration (vertical and horizontal black lines around points show 95% confidence interval for each polymorphism)

**Figure f5:**
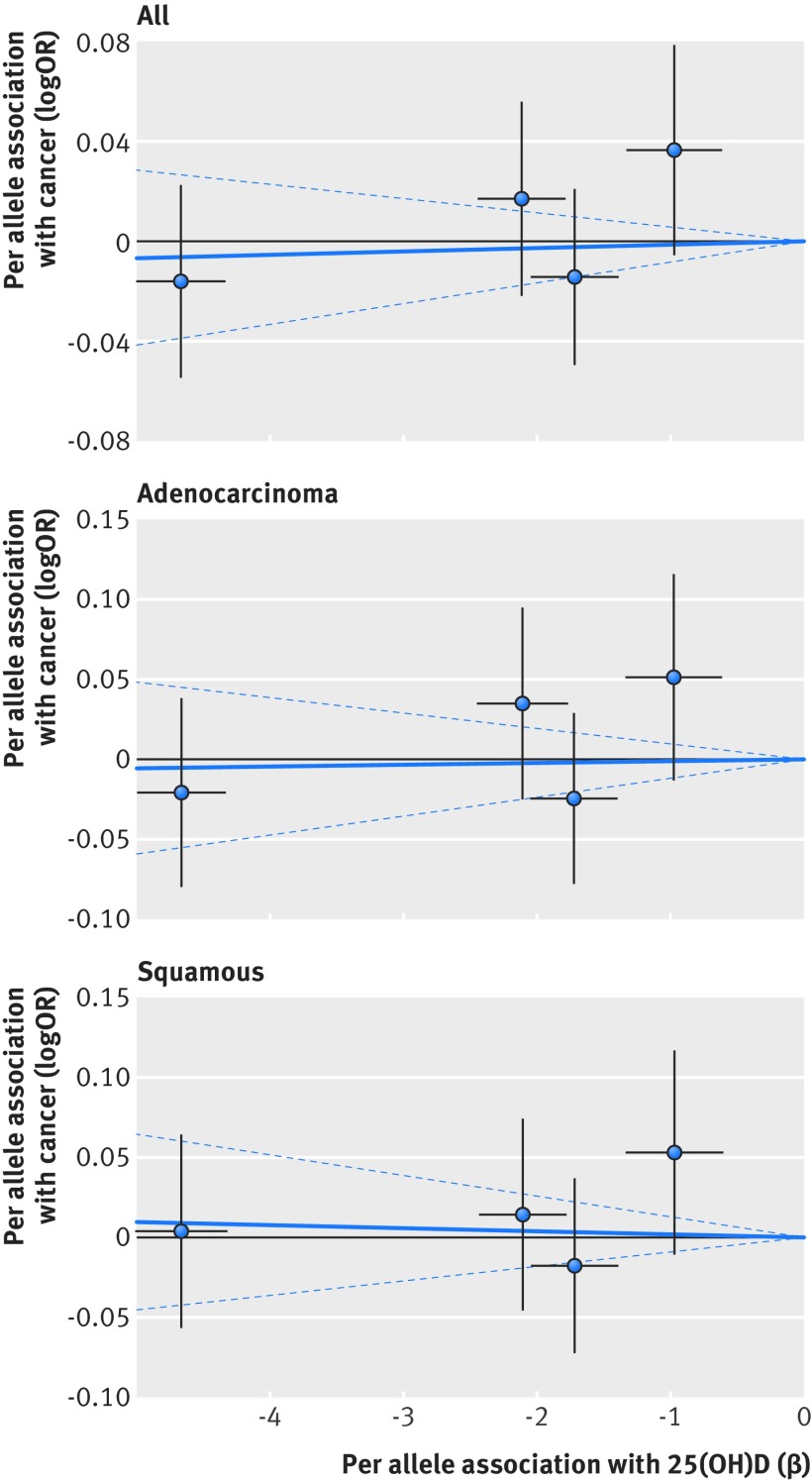
**Fig 5** Association between single nucleotide polymorphisms associated with vitamin D and risk of lung cancer and circulating 25(OH)D concentration. Per allele associations with risk plotted against per allele associations with continuous circulating 25(OH)D concentration (vertical and horizontal black lines around points show 95% confidence interval for each polymorphism)

**Figure f6:**
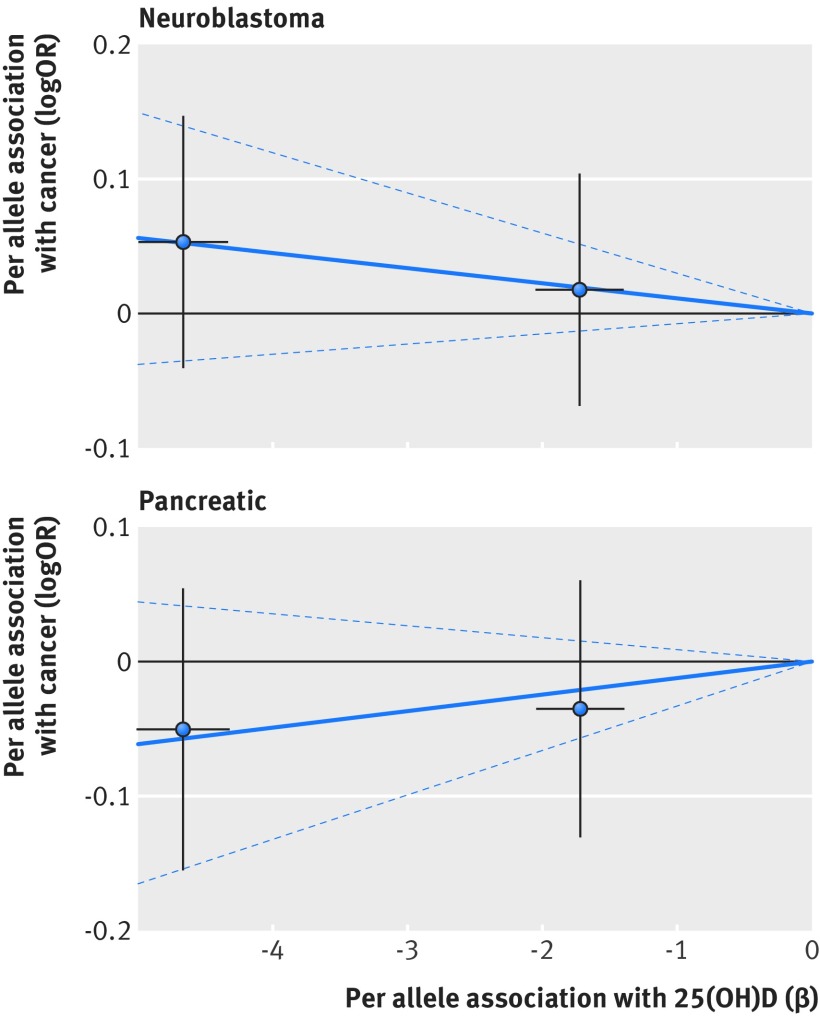
**Fig 6** Association between single nucleotide polymorphisms associated with vitamin D and risk of neuroblastoma and pancreatic cancer and circulating 25(OH)D concentration. Per allele associations with risk plotted against per allele associations with continuous circulating 25(OH)D concentration (vertical and horizontal black lines around points show 95% confidence interval for each polymorphism)

### Assessment of Mendelian randomisation assumptions

Mendelian randomisation estimates have a causal interpretation only if the instrumental variable assumptions of the method are valid. To satisfy the first assumption, we selected single nucleotide polymorphisms with a genome-wide significant association with 25(OH)D concentrations. We obtained estimates of association with continuous 25(OH)D concentration for each polymorphism from a previous large Mendelian randomisation study, which estimated that the *F* statistic was 230 (n=35 873) and 489 (n=38 191) for the vitamin D synthesis and metabolism allele scores, respectively.^28^

We carried out statistical tests and sensitivity analyses to evaluate the potential violation of the second and third assumptions. The goodness of fit tests indicated absence of horizontal pleiotropic effects of the four polymorphisms on cancer that are unrelated to the effect of each polymorphism on circulating 25(OH)D (table A in appendix 3). Over-identification tests also suggested that the effect estimates with different genetic variants were similar for all cancers. When we further evaluated presence of horizontal pleiotropy by performing the Mendelian randomisation analysis using two separate allelic scores (that is, vitamin D synthesis and metabolism), the results were identical and non-significant for all cancers (tables B and C in appendix 3). The MR-Egger regression method also did not show any evidence for the presence of horizontal pleiotropy for any of the reported associations (table D in appendix 3), as the P values for the intercept were large and the estimates adjusted for pleiotropy suggested null effects, although this method is expected to have low power to detect violation of assumptions when only four genetic instruments are used. The weighted median method also yielded no significant estimates (table D in appendix 3). We found no evidence in published genome-wide association studies that the four single nucleotide polymorphisms associated with vitamin D were genome-wide significantly associated with any other phenotype except 25(OH)D concentrations, which means that the third Mendelian randomisation assumption is probably not violated. Additionally, previous Mendelian randomisation studies using individual level data found no evidence for association between the vitamin D polymorphisms and potential environmental confounders.^9^ ^42^ ^43^

## Discussion

### Main findings and comparisons with the literature

In this large Mendelian randomisation study, we observed little evidence that a multi-single nucleotide polymorphism score for circulating 25(OH)D concentration was associated with risk of several cancers, including colorectal, breast, prostate, ovarian, lung, and pancreatic cancer and neuroblastoma or some of their subtypes. This was the first study with sufficient sample size under Mendelian randomisation assumptions to show a lack of causal effect for a linear association between 25(OH)D concentration and risk of these cancers.

#### Colorectal cancer

The overall evidence for an association between vitamin D and risk of specific cancers is mixed. Higher circulating 25(OH)D concentration has been associated with a lower risk of colorectal cancer. A systematic review of eight prospective studies that included 2690 cases of colorectal cancer observed a 34% (odds ratio 0.66, 95% confidence interval 0.54 to 0.81) lower risk of colorectal cancer for the top compared with the bottom quartile of 25(OH)D concentration.^2^ Another meta-analysis found that a 25 nmol/L increment in circulating 25(OH)D was associated with a relative risk of 0.74 (0.63 to 0.89).^37^ Although the current Mendelian randomisation study was powered for minimum detectable odds ratios up to 0.85 per 25 nmol/L in circulating 25(OH)D, it did not support such an association. In agreement with our findings, a previous Mendelian randomisation analysis of a Scottish case-control study of 2001 cases and 2237 controls did not find an association between genetically determined vitamin D concentrations and risk of colorectal cancer.^42^ The GECCO consortium found no association between the four single nucleotide polymorphisms associated with vitamin D and risk of colorectal cancer.^44^

#### Breast cancer

The prospective epidemiological evidence for an association between circulating 25(OH)D concentrations and risk of breast cancer is inconclusive. A meta-analysis by Gandini and colleagues reported that a 25 nmol/L increment in circulating 25(OH)D concentration was associated with a relative risk of 0.89 (95% confidence interval 0.81 to 0.98).^38^ Two subsequent meta-analyses observed no association between 25(OH)D concentration and risk of premenopausal breast cancer, whereas an inverse association was suggested for postmenopausal breast cancer.^45^ ^46^ Specifically, Bauer and colleagues suggested a borderline significant inverse association for postmenopausal women with a relative risk per 12.5 nmol/L****of circulating 25(OH)D of 0.97 (0.93 to 1.00).^45^ We did not find an association between genetically determined 25(OH)D concentrations and risk of breast cancer, and our study was powered to find minimum detectable odds ratios ranging from 0.84 to 0.87 per 25 nmol/L in 25(OH)D. Information on menopausal status was not available in the large genetic networks that we used, but most women in our sample had postmenopausal breast cancer. In agreement with our findings, the Women’s Health Initiative trial of vitamin D plus calcium supplementation in postmenopausal women did not support a protective association with breast cancer (hazard ratio 0.96, 95% confidence interval 0.85 to 1.09).^47^ A large cohort consortium of 9456 cases and 10 816 controls also found no association between the four single nucleotide polymorphisms associated with vitamin D and risk of breast cancer.^48^

#### Prostate cancer

A meta-analysis of 14 prospective studies published in 2011 provided little evidence that 25(OH)D concentration was associated with risk of total (odds ratio per 25 nmol/L), 1.04, 95% confidence interval 0.99 to 1.10) or aggressive (0.98, 0.84 to 1.15) prostate cancer.^39^ More recent prospective studies have reported null associations between circulating 25(OH)D concentration and risk of total prostate cancer, but inverse associations for aggressive or lethal disease.^49^ ^50^ Other prospective studies have reported positive associations for total disease and null associations for lethal disease,^51^ ^52^ or a significant U shaped association for total and aggressive disease^53^; whereas, a meta-analysis of 17 prospective studies published in 2014 observed a significantly increased risk of total prostate cancer (relative risk 1.18, 95% confidence interval 1.07 to 1.30) for the highest compared with the lowest concentrations of circulating 25(OH)D.^54^ A large cohort consortium of 10 018 cases of total prostate cancer and 11 052 controls found a significant association between a genetic risk score of the four single nucleotide polymorphisms associated with vitamin D and the risk of aggressive, but not total, prostate cancer.^55^ Our Mendelian randomisation analysis of 22 898 cases and 23 054 controls found no strong evidence for an association between genetically determined circulating 25(OH)D concentrations and risk of total or aggressive prostate cancer.

#### Ovarian cancer

Few prospective epidemiological studies have examined the association between circulating 25(OH)D concentrations and risk of ovarian cancer, and most have yielded null results.^40^ ^56^ A recent Mendelian randomisation study by Ong and colleagues observed a significant decrease (odds ratio 0.988, 95% confidence interval 0.979 to 0.997) in risk of ovarian cancer per 1 nmol/L increase in 25(OH)D concentrations,^57^ which translates to an odds ratio of 1.27 (1.06 to 1.51) per 20 nmol/L decrease in 25(OH)D. This study was twice the size of our Mendelian randomisation study for the gene-outcome associations but used three instead of four single nucleotide polymorphisms.^57^ Additionally, the authors used a published estimate for the association between rs2282679 and 25(OH)D concentrations from a small cohort of 2347 participants,^43^ whereas our analysis used published estimates from a large meta-analysis of about 38 000 participants.^28^ We did not find a significant association (odds ratio per unit increase in 25(OH)D, 1.005, 0.994 to 1.016). When we re-ran the Mendelian randomisation study using three (rs2282679, rs10741657, rs12785878) instead of four polymorphisms or using the same estimate for the association between rs2282679 with 25(OH)D concentrations as in the paper by Ong and colleagues, we observed almost identical non-significant results. Therefore, the small difference between the two Mendelian randomisation studies is plausible and can be explained by the larger statistical power of the gene-outcome association in the previous study.

#### Lung and pancreatic cancer and neuroblastoma

There is limited epidemiological evidence for a role of vitamin D in risk of lung and pancreatic cancer and neuroblastoma. A meta-analysis of 10 prospective studies reported a significant reduction (relative risk 0.95, 95% confidence interval 0.91 to 0.99) in risk of lung cancer for each 10 nmol/L increment in 25(OH)D concentration, but the heterogeneity between studies was large and a potential non-linear relation was suggested.^41^ We did not observe a significant association between genetically determined 25(OH)D concentrations and risk of lung or pancreatic cancer or neuroblastoma in the current Mendelian randomisation study, but our study was not powered to detect the small effect sizes suggested by the published meta-analysis for lung cancer.

### Strengths and limitations of this study

The main benefit of Mendelian randomisation studies is that they avoid biases that are commonly present in conventional observational literature. Resulting estimates have a causal interpretation only if the assumptions of the method hold. Though it is not possible to prove the validity of the assumptions, we performed sensitivity analyses and used several statistical tests to look for potential violations. We found no evidence of violation, though some of the statistical tests have low power to detect this when few genetic instruments are used (for example, MR-Egger).^34^ Previous Mendelian randomisation studies on vitamin D and risk of cancer or death that used individual level data, however, also did not suggest any violation of assumptions.^9^ ^42^ We used summary data for seven cancers and several of their subtypes, using thousands of cases of cancer and controls from several large genetic consortiums and published genome-wide association studies. We were powered to detect effect sizes of moderate magnitude for most primary cancer outcomes, but we cannot exclude the existence of causal clinically relevant effects of low magnitude.

Several limitations should be also considered in interpreting our findings. The summary level data that we used did not allow for stratified analyses by covariates of interest, such as age, sex, menopausal status, smoking, body mass index (BMI), and use of hormone replacement therapy or by other related genes or according to whether populations were vitamin D deficient or not. In addition, we could not explore potential non-linear associations between 25(OH)D concentrations and risk of cancer, which have been suggested by some studies. Furthermore, the currently known single nucleotide polymorphisms associated with vitamin D account for only a small amount of the variance observed in 25(OH)D concentration, but previous Mendelian randomisation studies have identified significant associations between vitamin D and several outcomes.^9^ ^43^ ^57^ ^58^ In addition, these single nucleotide polymorphisms do not predict concentrations of 1,25-dihydroxyvitamin D, which is the most biologically active metabolite of vitamin D, and also cannot predict vitamin D concentrations at the cellular level. Therefore, our results cannot be considered definitive. Future large pooling consortiums, larger genome-wide association studies of 25(OH)D concentration, and Mendelian randomisation studies with individual level data could deal with the latter issues. Moreover, large scale, general population, high dose vitamin D supplementation trials designed to overcome many of the limitations of previous trials (such as modest size, inadequate dose, relatively short duration, and small number of cancers) are ongoing^59^ ^60^ and might provide an improved understanding on the role of supplementation for development and death from non-skeletal outcomes.

### Conclusion

In summary, using a comprehensive Mendelian randomisation study, we found little evidence for linear causal associations between genetic determinants of circulating vitamin D concentration and risk of colorectal, breast, prostate, ovarian, lung, and pancreatic cancer and neuroblastoma, but we cannot rule out the existence of causal clinically relevant effects of low magnitude. Our results, in combination with previous literature, provide evidence that population-wide screening for vitamin D deficiency and subsequent widespread vitamin D supplementation should not currently be recommended as a strategy for primary cancer prevention.

**What is already known on this topic**

There is debate about whether vitamin D status is linked with disease or is just a correlate marker of overall health
Evidence from in vitro and animal model studies supports an anti-neoplastic role of vitamin D, but epidemiological studies and randomised controlled trials have yielded mixed results

**What this study adds**

This Mendelian randomisation study provides little evidence of a linear causal association between circulating vitamin D concentration and risk of colorectal, breast, prostate, ovarian, lung, and pancreatic cancer and neuroblastoma, but the existence of causal clinically relevant effects of low magnitude cannot be ruled out
Population-wide screening for vitamin D deficiency and subsequent widespread vitamin D supplementation cannot currently be recommended as a strategy for primary cancer prevention

## Web Extra

Extra material supplied by the author

Appendix 1: Supplementary methods

Appendix 2: Forest plots of associations

Appendix 3: Supplementary tables

Appendix 4: Further details of contributing consortiums, funding, and acknowledgments

## Notes

Members of the collaboration: Vasiliki I Dimitrakopoulou (Department of Hygiene and Epidemiology, School of Medicine, University of Ioannina, Ioannina, Greece; School of Mathematics and Statistics, University College Dublin, Dublin, Ireland); Konstantinos K Tsilidis (Department of Hygiene and Epidemiology, School of Medicine, University of Ioannina, Ioannina, Greece; Department of Epidemiology and Biostatistics, School of Public Health, Imperial College London, London, UK); Philip C Haycock and Sarah J Lewis (School of Social and Community Medicine and MRC Integrative Epidemiology Unit, University of Bristol, Bristol, UK); Niki L Dimou (Department of Hygiene and Epidemiology, School of Medicine, University of Ioannina, Ioannina, Greece); Kawthar Al-Dabhani (Department of Epidemiology and Biostatistics, School of Public Health, Imperial College London, London, UK); Richard M Martin (School of Social and Community Medicine and MRC Integrative Epidemiology Unit, University of Bristol, Bristol; National Institute for Health Research (NIHR) Bristol Nutritional Biomedical Research Unit, University Hospitals Bristol NHS Foundation Trust and the University of Bristol, Bristol, UK); Marc J Gunter and Paul Brennan (International Agency for Research on Cancer, Lyon, France); Alison Mondul (Department of Epidemiology, University of Michigan School of Public Health, Ann Arbor, MI, US); Irene M Shui (Department of Epidemiology, Harvard School of Public Health, Boston, MA, US); Evropi Theodoratou (Centre of Global Health Research, Usher Institute for Population Health Sciences and Informatics, University of Edinburg, UK); Katharina Nimptsch (Molecular Epidemiology Research Group, Max Delbrück Center for Molecular Medicine (MDC), Berlin, Germany); Sara Lindström (Department of Epidemiology, University of Washington, Seattle, WA, US); Demetrius Albanes and Sonja I Berndt (Division of Cancer Epidemiology and Genetics, National Cancer Institute, Bethesda, MD, US); Tilman Kühn (Division of Cancer Epidemiology, German Cancer Research Center (DKFZ), Heidelberg, Germany); Timothy J Key and Ruth C Travis (Cancer Epidemiology Unit, Nuffield Department of Population Health, University of Oxford, Oxford, UK); Leonid Raskin (Epidemiology Center, Department of Medicine, Vanderbilt-Ingram Cancer Center, Vanderbilt University, Nashville, TN, US); Andrew T Chan (Massachusetts General Hospital, Clinical and Translational Epidemiology Unit and Massachusetts General Hospital, Boston; Division of Gastroenterology, Boston; Brigham and Women’s Hospital, Channing Division of Network Medicine, Boston, MA, US); Mingyang Song (Massachusetts General Hospital, Clinical and Translational Epidemiology Unit and Division of Gastroenterology, Boston, MA; Harvard T.H. Chan School of Public Health, Departments of Nutrition and Epidemiology, Boston, MA, US); Amit D Joshi (Program in Genetic Epidemiology and Statistical Genetics, Department of Epidemiology, Harvard School of Public Health, Boston, MA, US); Yong-Bing Xiang (SKLORG & Department of Epidemiology, Shanghai Cancer Institute, Renji Hospital, Shanghai Jiaotong University School of Medicine, Shanghai, China); Sun Ha Jee (Graduate School of Public Health, Yonsei University, Seoul, Korea); Cornelia M Ulrich (Huntsman Cancer Institute and Department of Population Health Sciences, University of Utah School of Medicine, Salt Lake City, UT, US); John A Baron (Department of Medicine, School of Medicine, University of North Carolina, Chapel Hill, NC, USA); Graham G Giles (Cancer Epidemiology & Intelligence Division, Cancer Council Victoria, Melbourne, Australia; Centre for Epidemiology and Biostatistics, Melbourne School of Population and Global Health, University of Melbourne; Department of Epidemiology and Preventive Medicine, Monash University, Melbourne, Vic, Australia); Robert J MacInnis (Cancer Epidemiology & Intelligence Division, Cancer Council Victoria, Melbourne; Centre for Epidemiology and Biostatistics, Melbourne School of Population and Global Health, University of Melbourne, Vic, Australia); Melissa C Southey (Genetic Epidemiology Laboratory, Department of Pathology, University of Melbourne, Melbourne, Australia); Jane C Figueiredo (Department of Preventive Medicine, Keck School Of Medicine, University of Southern California; Samuel Oschin Comprehensive Cancer Institute, Cedars-Sinai Medical Center, Los Angeles, CA, US); Li Li (Department of Family Medicine and Community Health, Case Western Reserve University, Cleveland, OH, US); Christopher A Haiman (Department of Preventive Medicine, Keck School Of Medicine, Division of Cancer Epidemiology and Genetics, National Cancer Institute, Bethesda, MD, US); Victoria Stevens (Epidemiology Research Program, American Cancer Society, Atlanta, GA, US); Neil Caporaso (Division of Cancer Epidemiology and Genetics, National Cancer Institute, Bethesda, MD, US); Christopher I Amos, Department of Biomedical Data Sciences, Geisel School of Medicine at Dartmouth, Lebanon, NH, US); Xifeng Wu (University of Texas MD Anderson Cancer Center, Houston, TX, US); Rayjean J Hung (Lunenfeld-Tanenbaum Research Institute, Mount Sinai Hospital, Toronto, ON, Canada); Heike Bickeböller (Department of Genetic Epidemiology, University Medical Centre, Georg-August-Universität Göttingen, Göttingen, Germany); Angela Risch (Division of Cancer Genetics/Epigenetics, Department of Molecular Biology, University of Salzburg, Salzburg, Austria; Division of Epigenomics and Cancer Risk Factors, German Cancer Research Center (DKFZ), Heidelberg, Germany; Translational Lung Research Centre Heidelberg, German Center for Lung Research (DZL), Heidelberg, Germany); H-Erich Wichmann (Institute of Medical Informatics, Biometry and Epidemiology, Ludwig Maximilians University, Munich; Helmholtz Center Munich, Institute of Epidemiology II; Institute of Medical Statistics and Epidemiology, Technical University Munich, Germany); Richard Houlston (Division of Genetics and Epidemiology, Institute of Cancer Research, London, UK); Barbara L Banbury (Fred Hutchinson Cancer Research Center, Seattle, WA, US); Stephane Bezieau (Service de Génétique Médicale, Centre Hospitalier Universitaire (CHU) Nantes, Nantes, France); Hermann Brenner (Division of Clinical Epidemiology and Aging Research, German Cancer Research Center (DKFZ), Heidelberg; Division of Preventive Oncology, German Cancer Research Center (DKFZ) and National Center for Tumor Diseases (NCT), Heidelberg; German Cancer Consortium (DKTK), German Cancer Research Center (DKFZ), Heidelberg, Germany); Peter T Campbell (Epidemiology Research Program, American Cancer Society, Atlanta, GA, US); Graham Casey (Department of Public Health Sciences, University of Virginia, Charlottesville, VA, US); Jenny Chang-Claude (Division of Cancer Epidemiology, German Cancer Research Center (DKFZ), Heidelberg; Genetic Cancer Epidemiology, University Cancer Center Hamburg, University Medical Center Hamburg-Eppendorf, Hamburg, Germany); Steven J Gallinger (Department of Surgery, Mount Sinai Hospital, TN, Canada); Michael Hoffmeister (Division of Clinical Epidemiology and Aging Research, German Cancer Research Center (DKFZ), Heidelberg, Germany); Mark A Jenkins (Centre for Epidemiology and Biostatistics, Melbourne School of Population and Global Health, University of Melbourne, Vic, Australia); Loic Le Marchand (Epidemiology Program, University of Hawaii Cancer Center, Honololu, HI, US); Polly A Newcomb, Ulrike Peters, and Emily White (Fred Hutchinson Cancer Research Center, Seattle; Department of Epidemiology, University of Washington School of Public Health, Seattle, WA, USA); Martha L Slattery (Department of Internal Medicine, University of Utah Health Sciences Center, Salt Lake City, UT, US); Karani Santhanakrishnan Vimaleswaran (Department of Food and Nutritional Sciences, Hugh Sinclair Unit of Human Nutrition and Institute for Cardiovascular and Metabolic Research (ICMR), University of Reading, Reading, UK); Habibul Ahsan and Brandon L Pierce (Department of Public Health Sciences and Comprehensive Cancer Center and Department of Human Genetics, University of Chicago, Chicago, IL, US); the PRACTICAL Consortium (see appendix 4 for members from the Prostate Cancer Association Group to Investigate Cancer Associated Alterations in the Genome (PRACTICAL) consortium. Information of the consortium can be found at http://practical.ccge.medschl.cam.ac.uk/ ); Peter Kraft (Program in Genetic Epidemiology and Statistical Genetics, Department of Epidemiology, Harvard School of Public Health, Boston, MA, US); Joellen M Schildkraut (Department of Public Health Sciences, University of Virginia, Charlottesville, VA, US).

Contributors: VID and KKT contributed equally in this paper. The study was conceived and designed by PK, BLP, JMS and KKT. The data were analysed by VID, KKT, PCH, NLD, and KA-D. The first draft of the manuscript was prepared by VID and KKT, which was edited with the input from the writing team (RMM, SJL, MJG, AM, IMS, ET, KN, SL, DA, TK, TJK, and RCT). All other authors provided the data and revised the manuscript critically for important intellectual content. All authors gave final approval of the version to be published and have contributed to the manuscript. VID and KKT are guarantors.

Ethical approval: Not required.

Data sharing: No additional data available.

Transparency: The lead authors affirm that the manuscript is an honest, accurate, and transparent account of the study being reported; that no important aspects of the study have been omitted; and that any discrepancies from the study as planned have been explained.
