# Supplementary material for: Circulating vitamin D concentration and risk of seven cancers: Mendelian randomisation study
Source: BMJ. 2017 Oct 31;359:j4761. doi: 10.1136/bmj.j4761 (PMC5666592; doi:10.1136/bmj.j4761)
Supplement: Supplementary file 1 — Appendix 1: Supplementary methods [file dimv039614.ww1.pdf]

**Appendix 1: Supplementary methods [posted as supplied by author]**

## Statistical Analysis

We employed two Mendelian randomization (MR) methods utilizing summary genetic data: an inverse-variance weighted (IVW) average of SNP-specific associations and a likelihood-based method[1 2]. Briefly, the IVW method is based on meta-analysis principles and estimates the association of the vitamin D multi-SNP score on cancer risk as a weighted average of the individual genetic associations, where the effect estimate and its standard error are given by the following equations:

$$\hat{\beta}_{IVW} = \frac{\sum_k X_k Y_k \sigma_{Y_K}^{-2}}{\sum_k X_k^2 \sigma_{Y_K}^{-2}}$$

$$SE(\hat{\beta}_{IVW}) = \sqrt{\frac{1}{\sum_k X_k^2 \sigma_{Y_K}^{-2}}}$$

where $X_k$ is the per-allele estimate of the association between the $k^{th}$ SNP on serum 25(OH)D concentrations, $Y_k$ is the per-allele estimate of the $k^{th}$ SNP on the log-odds ratio of cancer risk and $\sigma_{Y_K}$ is its corresponding standard error. The likelihood-based approach assumes a linear relationship between the risk factor and the outcome, the associations of which are jointly modeled under a bivariate normal distribution. Assuming a common causal effect for all genetic variants, maximum likelihood methodology is used to obtain the effect estimate of interest. An advantage of the likelihood-based approach over the IVW method is that it produces valid estimates even in situations where the G (gene)-X and G-Y associations are correlated (i.e. measured on same or overlapping samples) or when the G-X association is estimated imprecisely[1]. Analyses for the IVW method were performed in the statistical software *R*, while likelihood-based analyses were performed using a web-based software at

http://spark.rstudio.com/sb452/summarized/[1]. The statistical analysis for pancreatic cancer and neuroblastoma were conducted using the MR-Base platform[3 4].

To secure the non-violation of the first assumption of MR, we only considered SNPs that had a genome-wide significant association with circulating 25(OH)D. Hence, all four SNPs were used as genetic instruments for testing the associations of continuous 25(OH)D concentrations, whereas fewer SNPs were used for vitamin D insufficiency at different thresholds based on the reported p-values in the GWAS[5 6].

To visualize the MR results, we plotted the per-allele associations for each SNP with cancer risk against the per-allele associations with circulating 25(OH)D. For the second assumption to hold, we expect that a SNP's association with cancer needs to be proportional to its association with serum 25(OH)D, and therefore that the plotted points fall along a line that passes through the origin and has a slope equal to the MR estimate[1]. In the absence of causal effect, a slope of zero is expected, whereas a stronger causal association would correspond to a line with a steeper slope.

To assess potential violation of the second assumption, a goodness-of-fit test was performed examining the null hypothesis that the association of each SNP with cancer risk is proportional to its association with 25(OH)D. To further assess potential violation of the second assumption due to directional horizontal pleiotropic SNP effects, we employed the MR-Egger regression method, which is an adaptation of the Egger regression in a meta-analysis[7]. The p-value of the intercept is a valid test of directional horizontal pleiotropy, whereas the slope of the MR-Egger regression is the horizontal pleiotropy-adjusted causal effect estimate. We further used the weighted median method to diagnose and protect against invalid genetic instruments, as this approach can provide a consistent estimate of the causal effect even when up to 50% of the information contributing to the analysis comes from genetic variants that are invalid instruments[8]. To further evaluate the overall validity of MR, over-identification tests

were performed to assess the null hypothesis that effect estimates from multiple genetic variants are identical[9]. If the estimates from multiple variants are similar and thus the over-identification test does not reject them, this suggests that none are biased. In addition, we conducted two further MR analyses using two separate allelic scores: a vitamin D synthesis allele score formed by rs10741657 and rs12785878; and a metabolism allele score formed by rs2282679 and rs6013897. To assess potential violation of the third assumption, we evaluated whether the four vitamin D SNPs were genome-wide statistically significantly associated with any other phenotype except 25(OH)D concentrations in published GWAS[10]. Finally, we calculated statistical power to estimate the minimum detectable magnitudes of association for all cancers and their subtypes using the web-based tool at http://glimmer.rstudio.com/kn3in/mRnd/[11].


1. Burgess S, Butterworth A, Thompson SG. Mendelian randomization analysis with multiple genetic variants using summarized data. Genet Epidemiol 2013;**37**(7):658-65.
2. Dastani Z, Hivert MF, Timpson N, et al. Novel loci for adiponectin levels and their influence on type 2 diabetes and metabolic traits: a multi-ethnic meta-analysis of 45,891 individuals. PLoS Genet 2012;**8**(3):e1002607.
3. Choi YJ, Kim YH, Cho CH, et al. Circulating levels of vitamin D and colorectal adenoma: A case-control study and a meta-analysis. World J Gastroenterol 2015;**21**(29):8868-77.
4. Gibran Hemani JZ, Kaitlin H Wade, Charles Laurin, Benjamin Elsworth, Stephen Burgess, Jack Bowden, Ryan Langdon, Vanessa Tan, James Yarmolinsky, Hashem A. Shihab, Nicholas Timpson, David M Evans, Caroline Relton, Richard M Martin, George Davey Smith, Tom R Gaunt, Philip C Haycock, The MR-Base Collaboration. MR-Base: a platform for systematic causal inference across the phenome using billions of genetic associations.
5. Ahn J, Yu K, Stolzenberg-Solomon R, et al. Genome-wide association study of circulating vitamin D levels. Hum Mol Genet 2010;**19**(13):2739-45.
6. Wang TJ, Zhang F, Richards JB, et al. Common genetic determinants of vitamin D insufficiency: a genome-wide association study. Lancet 2010;**376**(9736):180-8.
7. Bowden J, Davey Smith G, Burgess S. Mendelian randomization with invalid instruments: effect estimation and bias detection through Egger regression. Int J Epidemiol 2015;**44**(2):512-25.
8. Bowden J, Davey Smith G, Haycock PC, et al. Consistent Estimation in Mendelian Randomization with Some Invalid Instruments Using a Weighted Median Estimator. Genet Epidemiol 2016;**40**(4):304-14.
9. Glymour MM, Tchetgen Tchetgen EJ, Robins JM. Credible Mendelian randomization studies: approaches for evaluating the instrumental variable assumptions. Am J Epidemiol 2012;**175**(4):332-9.
10. Welter D, MacArthur J, Morales J, et al. The NHGRI GWAS Catalog, a curated resource of SNP-trait associations. Nucleic acids research 2014;**42**(Database issue):D1001-6.
11. Brion MJ, Shakhbazov K, Visscher PM. Calculating statistical power in Mendelian randomization studies. Int J Epidemiol 2013;**42**(5):1497-501.
