# Supplementary material for: Circulating vitamin D concentration and risk of seven cancers: Mendelian randomisation study
Source: BMJ. 2017 Oct 31;359:j4761. doi: 10.1136/bmj.j4761 (PMC5666592; doi:10.1136/bmj.j4761)
Supplement: Supplementary file 2 — Appendix 2: Forest plots of associations [file dimv039614.ww2.pdf]

Appendix 2: Forest plots of associations between each 25(OH)D-associated SNP and cancer risk (horizontal lines indicating 95% confidence intervals).

[posted as supplied by author]

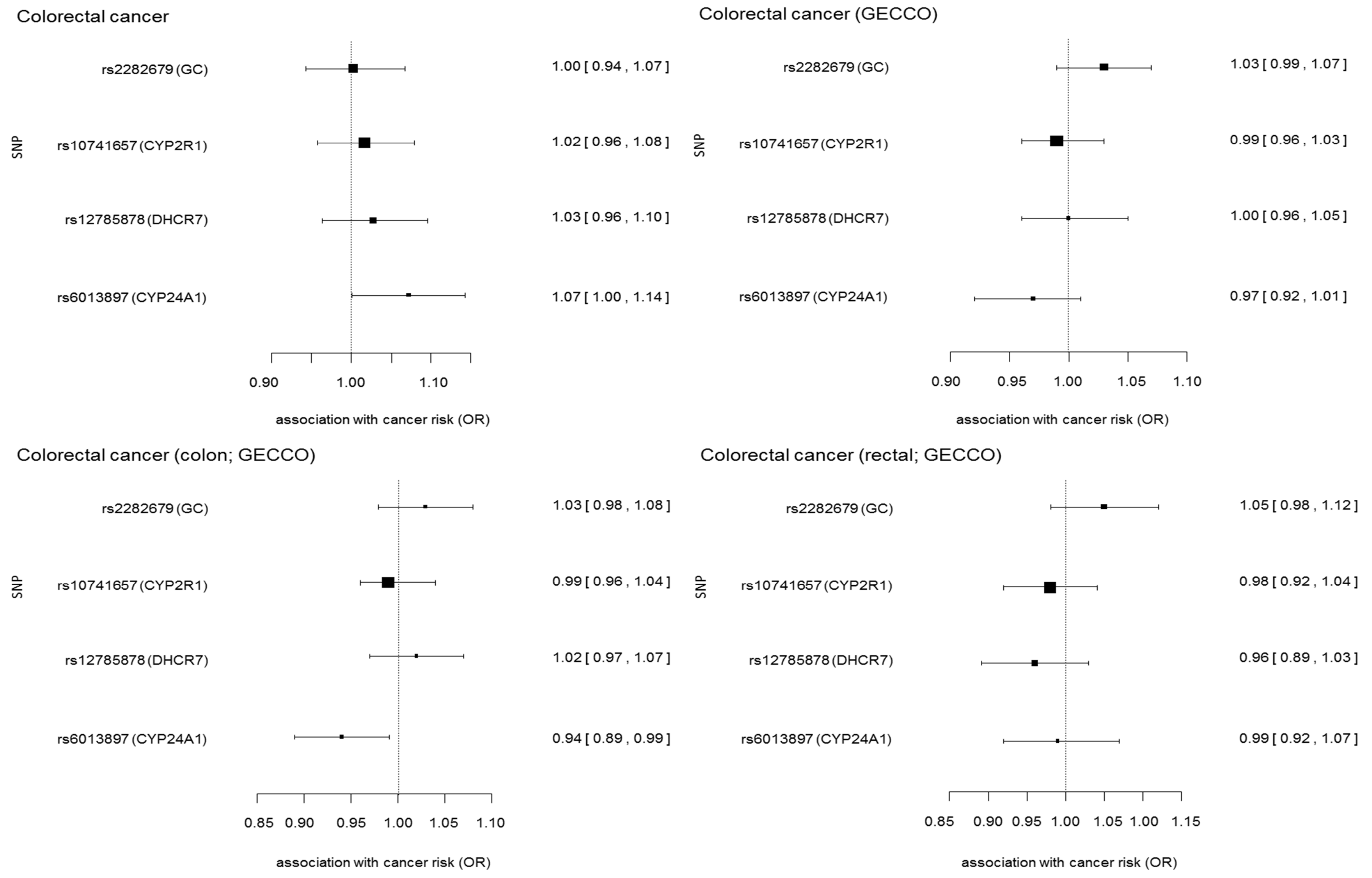

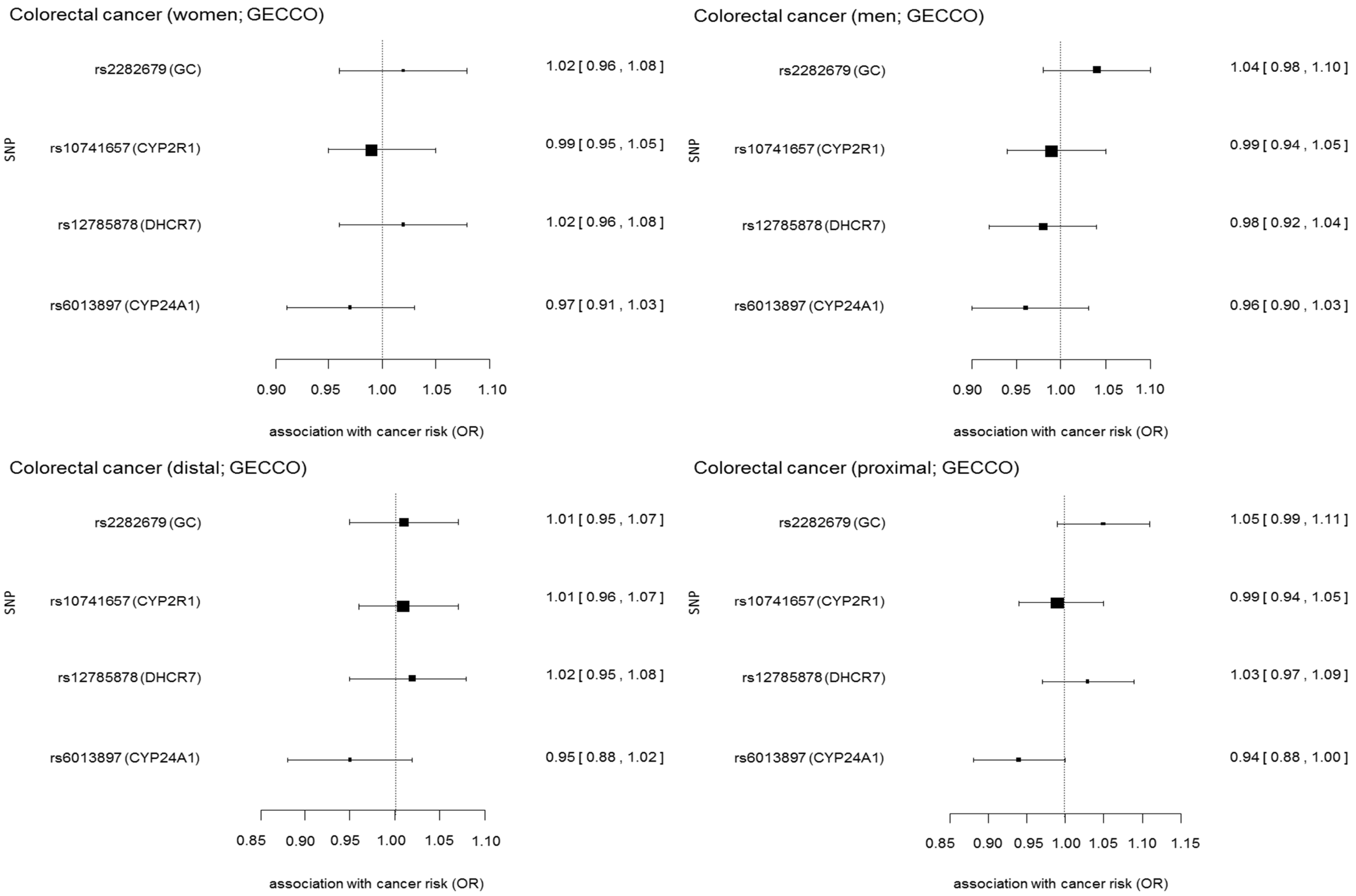
Colorectal cancer (women; GECCO)
SNP
rs2282679 (GC)
rs10741657 (CYP2R1)
rs12785878 (DHCR7)
rs6013897 (CYP24A1)
1.02 [0.96 , 1.08]
0.99 [0.95 , 1.05]
1.02 [0.96 , 1.08]
0.97 [0.91 , 1.03]
0.90 0.95 1.00 1.05 1.10
association with cancer risk (OR)
Colorectal cancer (men; GECCO)
SNP
rs2282679 (GC)
rs10741657 (CYP2R1)
rs12785878 (DHCR7)
rs6013897 (CYP24A1)
1.04 [0.98 , 1.10]
0.99 [0.94 , 1.05]
0.98 [0.92 , 1.04]
0.96 [0.90 , 1.03]
0.90 0.95 1.00 1.05 1.10
association with cancer risk (OR)
Colorectal cancer (distal; GECCO)
SNP
rs2282679 (GC)
rs10741657 (CYP2R1)
rs12785878 (DHCR7)
rs6013897 (CYP24A1)
1.01 [0.95 , 1.07]
1.01 [0.96 , 1.07]
1.02 [0.95 , 1.08]
0.95 [0.88 , 1.02]
0.85 0.90 0.95 1.00 1.05 1.10
association with cancer risk (OR)
Colorectal cancer (proximal; GECCO)
SNP
rs2282679 (GC)
rs10741657 (CYP2R1)
rs12785878 (DHCR7)
rs6013897 (CYP24A1)
1.05 [0.99 , 1.11]
0.99 [0.94 , 1.05]
1.03 [0.97 , 1.09]
0.94 [0.88 , 1.00]
0.85 0.90 0.95 1.00 1.05 1.10 1.15
association with cancer risk (OR)

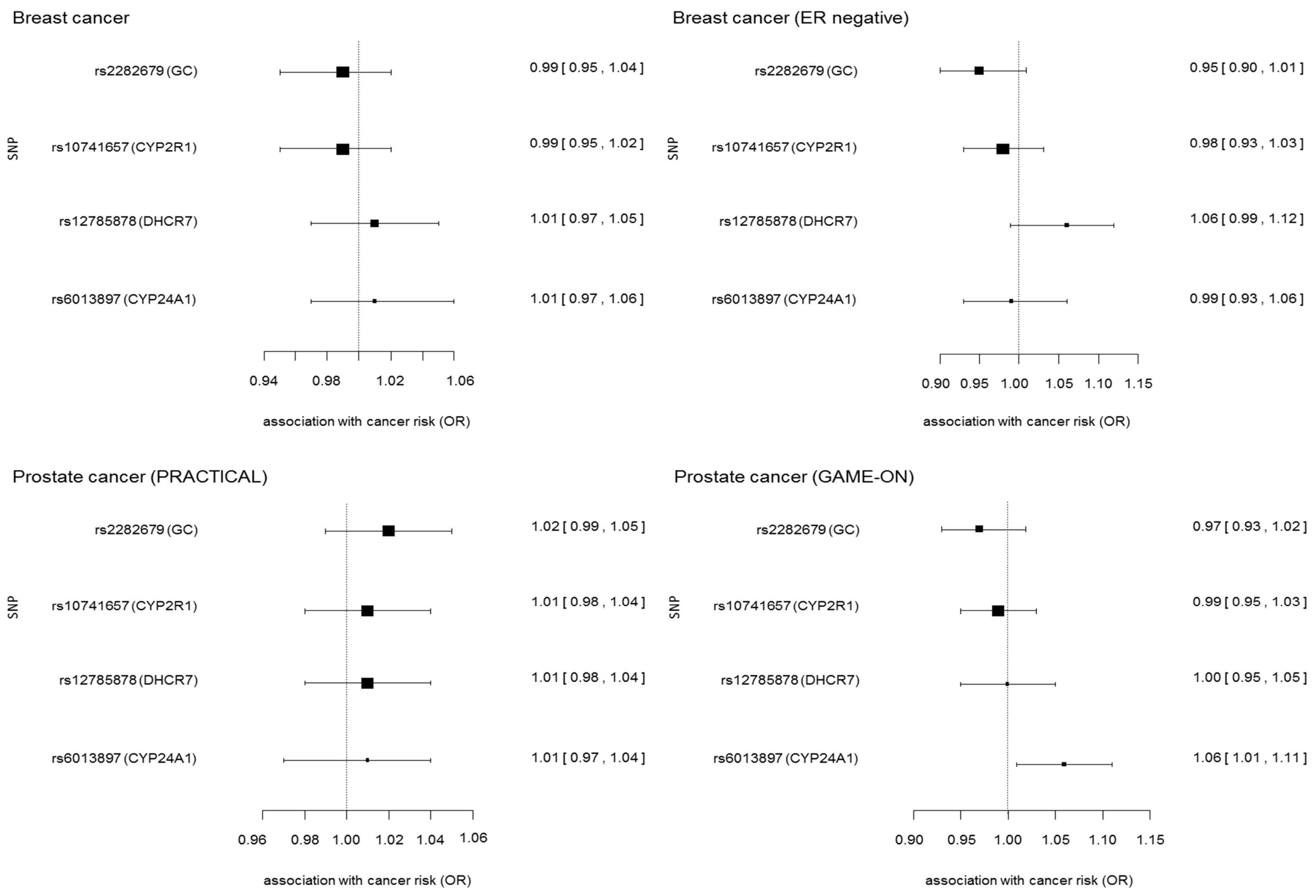
Breast cancer
SNP
rs2282679 (GC)
rs10741657 (CYP2R1)
rs12785878 (DHCR7)
rs6013897 (CYP24A1)
0.99 [0.95 , 1.04]
0.99 [0.95 , 1.02]
1.01 [0.97 , 1.05]
1.01 [0.97 , 1.06]
0.94 0.98 1.02 1.06
association with cancer risk (OR)
Breast cancer (ER negative)
SNP
rs2282679 (GC)
rs10741657 (CYP2R1)
rs12785878 (DHCR7)
rs6013897 (CYP24A1)
0.95 [0.90 , 1.01]
0.98 [0.93 , 1.03]
1.06 [0.99 , 1.12]
0.99 [0.93 , 1.06]
0.90 0.95 1.00 1.05 1.10 1.15
association with cancer risk (OR)
Prostate cancer (PRACTICAL)
SNP
rs2282679 (GC)
rs10741657 (CYP2R1)
rs12785878 (DHCR7)
rs6013897 (CYP24A1)
1.02 [0.99 , 1.05]
1.01 [0.98 , 1.04]
1.01 [0.98 , 1.04]
1.01 [0.97 , 1.04]
0.96 0.98 1.00 1.02 1.04 1.06
association with cancer risk (OR)
Prostate cancer (GAME-ON)
SNP
rs2282679 (GC)
rs10741657 (CYP2R1)
rs12785878 (DHCR7)
rs6013897 (CYP24A1)
0.97 [0.93 , 1.02]
0.99 [0.95 , 1.03]
1.00 [0.95 , 1.05]
1.06 [1.01 , 1.11]
0.90 0.95 1.00 1.05 1.10 1.15
association with cancer risk (OR)

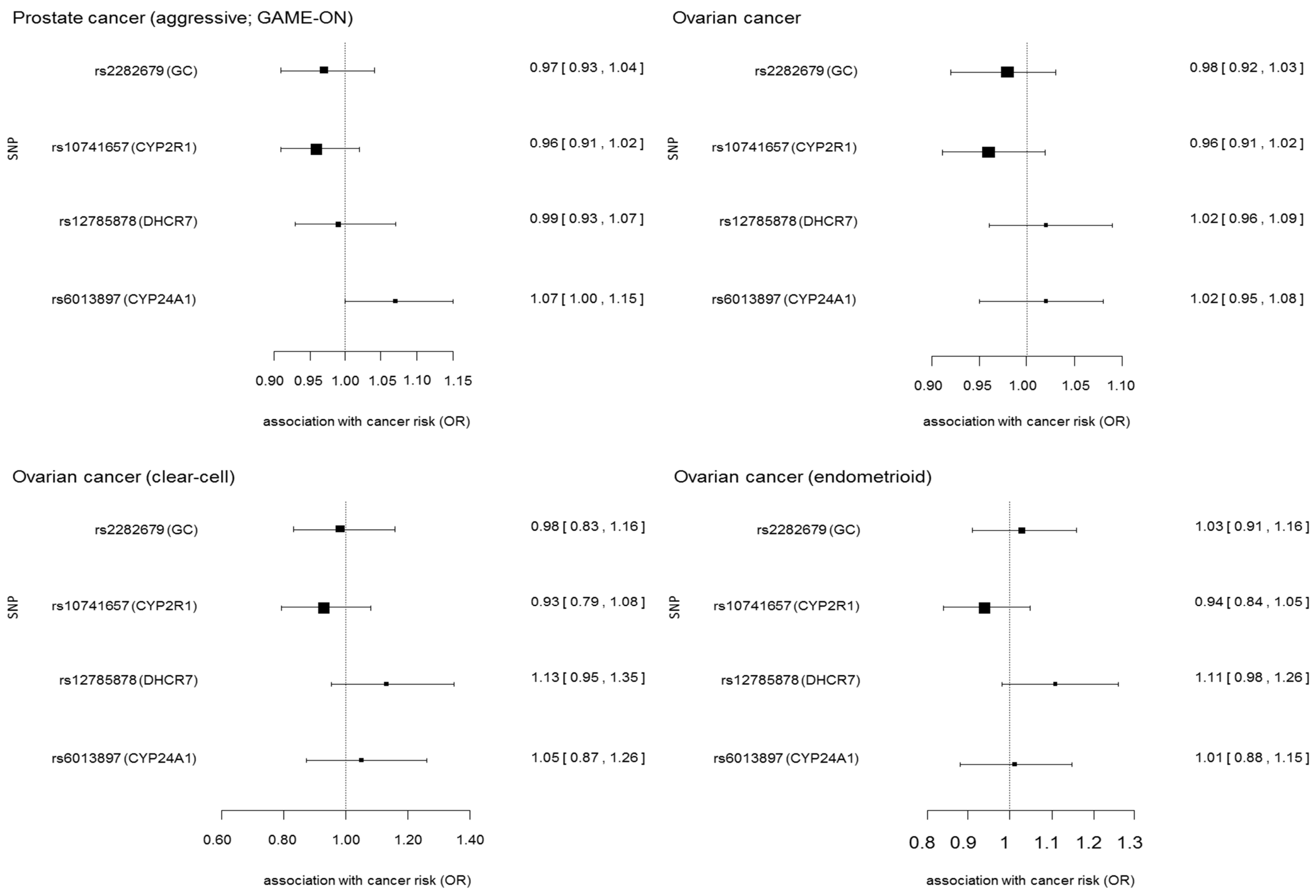
Prostate cancer (aggressive; GAME-ON)
SNP
rs2282679 (GC)
rs10741657 (CYP2R1)
rs12785878 (DHCR7)
rs6013897 (CYP24A1)
0.97 [0.93 , 1.04]
0.96 [0.91 , 1.02]
0.99 [0.93 , 1.07]
1.07 [1.00 , 1.15]
0.90 0.95 1.00 1.05 1.10 1.15
association with cancer risk (OR)
Ovarian cancer
SNP
rs2282679 (GC)
rs10741657 (CYP2R1)
rs12785878 (DHCR7)
rs6013897 (CYP24A1)
0.98 [0.92 , 1.03]
0.96 [0.91 , 1.02]
1.02 [0.96 , 1.09]
1.02 [0.95 , 1.08]
0.90 0.95 1.00 1.05 1.10
association with cancer risk (OR)
Ovarian cancer (clear-cell)
SNP
rs2282679 (GC)
rs10741657 (CYP2R1)
rs12785878 (DHCR7)
rs6013897 (CYP24A1)
0.98 [0.83 , 1.16]
0.93 [0.79 , 1.08]
1.13 [0.95 , 1.35]
1.05 [0.87 , 1.26]
0.60 0.80 1.00 1.20 1.40
association with cancer risk (OR)
Ovarian cancer (endometrioid)
SNP
rs2282679 (GC)
rs10741657 (CYP2R1)
rs12785878 (DHCR7)
rs6013897 (CYP24A1)
1.03 [0.91 , 1.16]
0.94 [0.84 , 1.05]
1.11 [0.98 , 1.26]
1.01 [0.88 , 1.15]
0.8 0.9 1 1.1 1.2 1.3
association with cancer risk (OR)

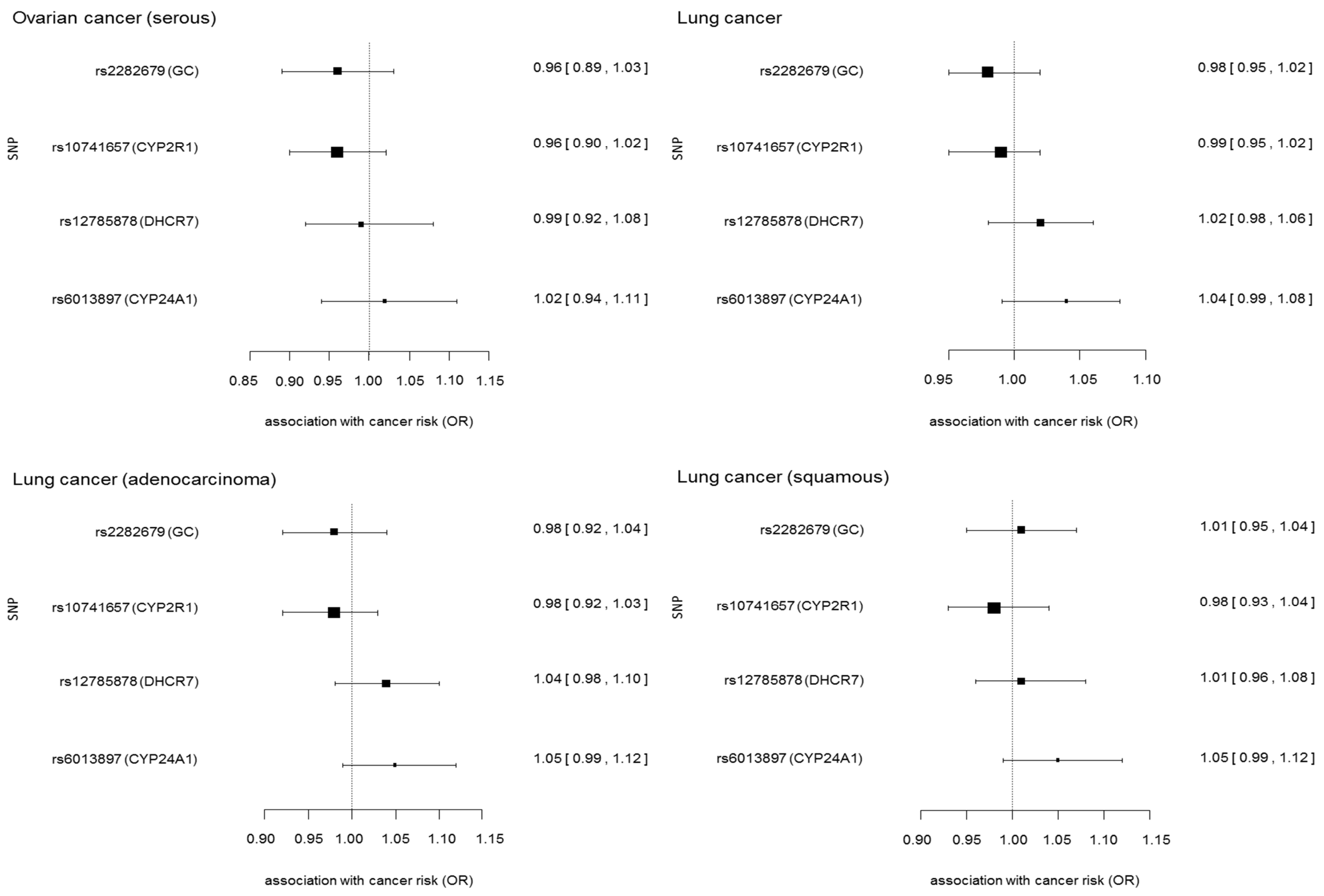
Ovarian cancer (serous)
SNP
rs2282679 (GC)
rs10741657 (CYP2R1)
rs12785878 (DHCR7)
rs6013897 (CYP24A1)
0.96 [0.89 , 1.03]
0.96 [0.90 , 1.02]
0.99 [0.92 , 1.08]
1.02 [0.94 , 1.11]
0.85 0.90 0.95 1.00 1.05 1.10 1.15
association with cancer risk (OR)
Lung cancer
SNP
rs2282679 (GC)
rs10741657 (CYP2R1)
rs12785878 (DHCR7)
rs6013897 (CYP24A1)
0.98 [0.95 , 1.02]
0.99 [0.95 , 1.02]
1.02 [0.98 , 1.06]
1.04 [0.99 , 1.08]
0.95 1.00 1.05 1.10
association with cancer risk (OR)
Lung cancer (adenocarcinoma)
SNP
rs2282679 (GC)
rs10741657 (CYP2R1)
rs12785878 (DHCR7)
rs6013897 (CYP24A1)
0.98 [0.92 , 1.04]
0.98 [0.92 , 1.03]
1.04 [0.98 , 1.10]
1.05 [0.99 , 1.12]
0.90 0.95 1.00 1.05 1.10 1.15
association with cancer risk (OR)
Lung cancer (squamous)
SNP
rs2282679 (GC)
rs10741657 (CYP2R1)
rs12785878 (DHCR7)
rs6013897 (CYP24A1)
1.01 [0.95 , 1.04]
0.98 [0.93 , 1.04]
1.01 [0.96 , 1.08]
1.05 [0.99 , 1.12]
0.90 0.95 1.00 1.05 1.10 1.15
association with cancer risk (OR)

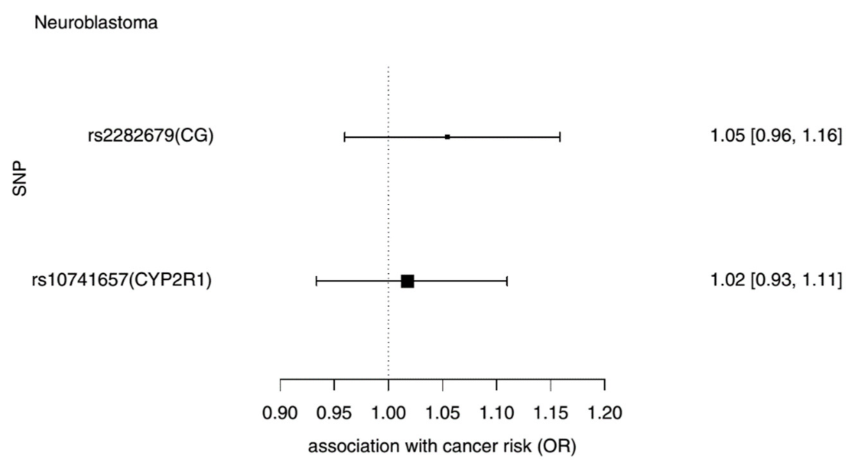
Neuroblastoma
SNP
rs2282679(CG)
1.05 [0.96, 1.16]
rs10741657(CYP2R1)
1.02 [0.93, 1.11]
0.90 0.95 1.00 1.05 1.10 1.15 1.20
association with cancer risk (OR)

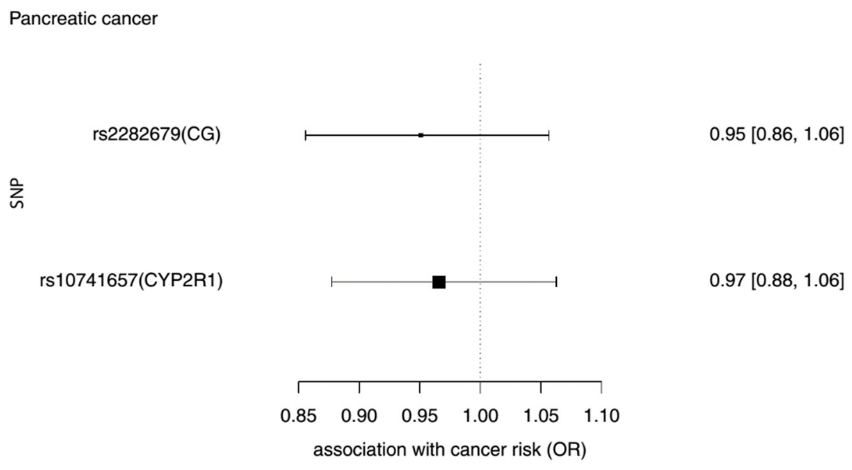
Pancreatic cancer
SNP
rs2282679(CG)
0.95 [0.86, 1.06]
rs10741657(CYP2R1)
0.97 [0.88, 1.06]
0.85 0.90 0.95 1.00 1.05 1.10
association with cancer risk (OR)
