# Supplementary material for: Circulating vitamin D concentration and risk of seven cancers: Mendelian randomisation study
Source: BMJ. 2017 Oct 31;359:j4761. doi: 10.1136/bmj.j4761 (PMC5666592; doi:10.1136/bmj.j4761)
Supplement: Supplementary file 3 — Appendix 3: Supplementary tables [file dimv039614.ww3.pdf]

**Appendix 3**: Supplementary tables [posted as supplied by author]

**Table A**: P-values for the goodness-of-fit test for continuous 25(OH)D and cancer risk before and after exclusion of SNPs.

| Cancer Type | P-value of goodness-of-fit test including all SNPs[a] | SNP excluded | P-value of the goodness-of-fit test after SNP exclusion |
|---|---|---|---|
| **Colorectal** | | | |
| All – GAME-ON | 0.20 | None | NA |
| All – GECCO | 0.36 | None | NA |
| All (women) – GECCO | 0.64 | None | NA |
| All (men) – GECCO | 0.45 | None | NA |
| Colon – GECCO | 0.11 | None | NA |
| Rectal – GECCO | 0.35 | None | NA |
| Distal Colon – GECCO | 0.43 | None | NA |
| Proximal Colon – GECCO | 0.13 | None | NA |
| **Breast** | | | |
| All | 0.72 | None | NA |
| ER-negative | 0.14 | None | NA |
| **Prostate** | | | |
| All – PRACTICAL | 0.99 | None | NA |
| All – GAME-ON | 0.08 | None | NA |
| Aggressive – GAME-ON | 0.12 | None | NA |
| **Ovarian** | | | |
| All | 0.52 | None | NA |
| Clear-cell | 0.38 | None | NA |
| Endometrioid | 0.28 | None | NA |
| Serous | 0.76 | None | NA |
| **Lung** | | | |
| All | 0.18 | None | NA |
| Adenocarcinoma | 0.17 | None | NA |
| Squamous | 0.38 | None | NA |
| **Pancreatic**[b] | 0.76 | None | NA |
| **Neuroblastoma**[b] | 0.97 | None | NA |

[a]This test assesses the potential violation of the second assumption of Mendelian randomization, and is performed by examining the null hypothesis that the association of each SNP with cancer risk is proportional to its association with 25(OH)D concentrations. When the null hypothesis was rejected, we repeated the analysis each time removing the SNP with the lowest significance, until the test was no longer statistically significant.

[b]All four SNPs were used for all cancers, except for pancreatic cancer and neuroblastoma, where only two SNPs (rs10741657, rs2282679) were available.

**Table B:** Mendelian randomization estimates between multi-SNP risk scores of continuous 25(OH)D synthesis (rs10741657, rs12785878) and cancer risk calculated using the inverse-variance weighted method (left) and the likelihood method (right).

| | Inverse-Variance Weighted Method | | | Likelihood Method | | |
|---|---|---|---|---|---|---|
| **Cancer Type** | **OR[a]** | **95% CI** | **p-value** | **OR[a]** | **95% CI** | **p-value** |
| **Colorectal** | | | | | | |
| All – GAME-ON | 1.33 | (0.75, 2.35) | 0.34 | 1.33 | (0.75, 2.35) | 0.34 |
| All – GECCO | 1.00 | (0.69, 1.45) | 0.99 | 1.00 | (0.69, 1.45) | 0.99 |
| All (women) – GECCO | 0.89 | (0.54, 1.48) | 0.67 | 0.89 | (0.54, 1.48) | 0.67 |
| All (men) – GECCO | 1.16 | (0.67, 1.99) | 0.61 | 1.16 | (0.67, 2.00) | 0.61 |
| Colon – GECCO | 0.88 | (0.58, 1.34) | 0.55 | 0.88 | (0.57, 1.34) | 0.55 |
| Rectal – GECCO | 1.50 | (0.80, 2.80) | 0.20 | 1.50 | (0.80, 2.81) | 0.21 |
| Distal Colon – GECCO | 0.84 | (0.48, 1.46) | 0.54 | 0.84 | (0.48, 1.46) | 0.54 |
| Proximal Colon – GECCO | 0.86 | (0.51, 1.43) | 0.56 | 0.86 | (0.51, 1.43) | 0.56 |
| **Breast** | | | | | | |
| All | 1.02 | (0.73, 1.43) | 0.91 | 1.02 | (0.73, 1.43) | 0.92 |
| ER-negative | 0.80 | (0.47, 1.36) | 0.42 | 0.80 | (0.47, 1.36) | 0.42 |
| **Prostate** | | | | | | |
| All – PRACTICAL | 0.88 | (0.67, 1.14) | 0.34 | 0.88 | (0.67, 1.14) | 0.34 |
| All – GAME-ON | 1.05 | (0.70, 1.57) | 0.82 | 1.05 | (0.70, 1.57) | 0.82 |
| Aggressive– GAME-ON | 1.32 | (0.74, 2.34) | 0.35 | 1.32 | (0.74, 2.34) | 0.35 |
| **Ovarian** | | | | | | |
| All | 1.12 | (0.66, 1.92) | 0.69 | 1.12 | (0.65, 1.93) | 0.69 |
| Clear-cell | 0.77 | (0.17, 3.44) | 0.74 | 0.77 | (0.17, 3.46) | 0.74 |
| Endometrioid | 0.73 | (0.25, 2.16) | 0.58 | 0.73 | (0.24, 2.17) | 0.58 |
| Serous | 1.38 | (0.71, 2.69) | 0.35 | 1.38 | (0.71, 2.69) | 0.35 |
| **Lung** | | | | | | |
| All | 0.98 | (0.70, 1.38) | 0.93 | 0.98 | (0.70, 1.38) | 0.93 |
| Adenocarcinoma | 0.93 | (0.55, 1.58) | 0.81 | 0.93 | (0.55, 1.58) | 0.81 |
| Squamous | 1.02 | (0.60, 1.73) | 0.95 | 1.02 | (0.60, 1.73) | 0.95 |
| **Pancreatic[b]** | 1.66 | (0.41, 4.00) | 0.47 | 1.66 | (0.41, 4.00) | 0.47 |
| **Neuroblastoma[b]** | 0.77 | (0.22, 2.70) | 0.69 | 0.77 | (0.22, 2.70) | 0.69 |

[a]The odds ratios (ORs) represent increase/decrease of risk per 25 nmol/L increase in the natural scale of 25(OH)D.

[b]Only rs10741657 was used in these analyses.

**Table C:** Mendelian randomization estimates between multi-SNP risk scores of continuous 25(OH)D metabolism (rs2282679, rs6013897) and cancer risk calculated using the inverse-variance weighted method (left) and the likelihood method (right).

| | Inverse-Variance Weighted Method | | | Likelihood Method | | |
|---|---|---|---|---|---|---|
| **Cancer Type** | **OR[a]** | **95% CI** | **p-value** | **OR[a]** | **95% CI** | **p-value** |
| **Colorectal** | | | | | | |
| All – GAME-ON | 0.96 | (0.69, 1.33) | 0.80 | 0.96 | (0.69, 1.33) | 0.80 |
| All – GECCO | 0.89 | (0.72, 1.10) | 0.28 | 0.89 | (0.72, 1.10) | 0.28 |
| All (women) – GECCO | 0.93 | (0.69, 1.24) | 0.63 | 0.93 | (0.69, 1.24) | 0.63 |
| All (men) – GECCO | 0.84 | (0.62, 1.16) | 0.30 | 0.84 | (0.62, 1.16) | 0.30 |
| Colon – GECCO | 0.91 | (0.71, 1.16) | 0.45 | 0.91 | (0.71, 1.16) | 0.45 |
| Rectal – GECCO | 0.79 | (0.56, 1.13) | 0.20 | 0.79 | (0.56, 1.13) | 0.20 |
| Distal Colon – GECCO | 1.02 | (0.74, 1.40) | 0.93 | 1.02 | (0.74, 1.40) | 0.93 |
| Proximal Colon – GECCO | 0.82 | (0.61, 1.10) | 0.18 | 0.81 | (0.61, 1.10) | 0.18 |
| **Breast** | | | | | | |
| All | 1.06 | (0.87, 1.29) | 0.58 | 1.06 | (0.87, 1.29) | 0.57 |
| ER-negative | 1.30 | (0.96, 1.76) | 0.09 | 1.30 | (0.96, 1.76) | 0.09 |
| **Prostate** | | | | | | |
| All – PRACTICAL | 0.89 | (0.76, 1.04) | 0.15 | 0.89 | (0.76, 1.04) | 0.15 |
| All – GAME-ON | 1.09 | (0.86, 1.39) | 0.48 | 1.09 | (0.86, 1.40) | 0.48 |
| Aggressive– GAME-ON | 1.09 | (0.77, 1.54) | 0.65 | 1.09 | (0.77, 1.54) | 0.65 |
| **Ovarian** | | | | | | |
| All | 1.13 | (0.83, 1.53) | 0.46 | 1.13 | (0.83, 1.53) | 0.46 |
| Clear-cell | 1.08 | (0.45, 2.60) | 0.88 | 1.08 | (0.45, 2.60) | 0.88 |
| Endometrioid | 0.87 | (0.46, 1.63) | 0.67 | 0.87 | (0.46, 1.63) | 0.67 |
| Serous | 1.23 | (0.84, 1.80) | 0.30 | 1.23 | (0.84, 1.80) | 0.30 |
| **Lung** | | | | | | |
| All | 1.05 | (0.86, 1.29) | 0.63 | 1.05 | (0.86, 1.29) | 0.63 |
| Adenocarcinoma | 1.07 | (0.78, 1.46) | 0.70 | 1.07 | (0.78, 1.46) | 0.70 |
| Squamous | 0.93 | (0.68, 1.28) | 0.67 | 0.93 | (0.67, 1.28) | 0.67 |
| **Pancreatic[b]** | 1.31 | (0.75, 2.33) | 0.35 | 1.31 | (0.75, 2.33) | 0.35 |
| **Neuroblastoma[b]** | 0.75 | (0.45, 1.25) | 0.27 | 0.75 | (0.45, 1.25) | 0.27 |

[a]The odds ratios (ORs) represent increase/decrease of risk per 25 nmol/L increase in the natural scale of 25(OH)D.

[b]Only rs2282679 was used in these analyses.

**Table D:** Mendelian randomization estimates between multi-SNP risk scores of continuous 25(OH)D and cancer risk calculated using Egger's regression and weighted median approach[a].

| | MR Egger | | Weighted median |
|---|---|---|---|
| **Cancer Type** | **Intercept p-value** | **Slope OR[c] (95% CI)** | **OR[c] (95% CI)** |
| **Colorectal** | | | |
| All – GAME-ON | 0.67 | 1.24 (0.70, 2.21) | 1.08 (0.79, 1.46) |
| All – GECCO | 0.25 | 0.70 (0.49, 1.02) | 0.89 (0.73, 1.08) |
| All (women) – GECCO | 0.51 | 0.77 (0.46, 1.28) | 0.90 (0.68, 1.18) |
| All (men) – GECCO | 0.27 | 0.63 (0.36, 1.10) | 0.88 (0.66, 1.18) |
| Colon – GECCO | 0.33 | 0.66 (0.42, 1.02) | 0.86 (0.69, 1.08) |
| Rectal – GECCO | 0.28 | 0.62 (0.33, 1.14) | 0.91 (0.65, 1.28) |
| Distal Colon – GECCO | 0.59 | 0.82 (0.46, 1.44) | 0.93 (0.69, 1.26) |
| Proximal Colon – GECCO | 0.25 | 0.55 (0.32, 0.92) | 0.77 (0.58, 1.02) |
| **Breast** | | | |
| All | 0.66 | 1.13 (0.80, 1.60) | 1.06 (0.88, 1.27) |
| ER-negative | 0.65 | 1.39 (0.82, 2.40) | 1.30 (0.97, 1.75) |
| **Prostate** | | | |
| All – PRACTICAL | 0.94 | 0.90 (0.67, 1.19) | 0.89 (0.77, 1.03) |
| All – GAME-ON | 0.24 | 1.55 (1.02, 2.40) | 1.15 (0.92, 1.44) |
| Aggressive – GAME-ON | 0.55 | 1.52 (0.82, 2.78) | 1.17 (0.84, 1.61) |
| **Ovarian** | | | |
| All | 0.86 | 1.18 (0.68, 2.05) | 1.12 (0.83, 1.51) |
| Clear-cell | 0.78 | 1.28 (0.27, 6.22) | 1.03 (0.44, 2.42) |
| Endometrioid | 0.95 | 0.79 (0.25, 2.49) | 0.87 (0.46, 1.61) |
| Serous | 0.93 | 1.30 (0.67, 2.58) | 1.24 (0.87, 1.78) |
| **Lung** | | | |
| All | 0.38 | 1.28 (0.90, 1.85) | 1.07 (0.88, 1.30) |
| Adenocarcinoma | 0.44 | 1.39 (0.80. 2.43) | 1.08 (0.80, 1.46) |
| Squamous | 0.54 | 1.16 (0.67, 2.03) | 0.97 (0.72, 1.30) |
| **Pancreatic[b]** | NA | NA | NA |
| **Neuroblastoma[b]** | NA | NA | NA |

[a]To further assess potential violation of the second assumption of Mendelian randomization (MR) due to pleiotropic SNP effects, we employed the MR-Egger regression method, which is an adaptation of the Egger regression in a meta-analysis. The p-value of the intercept is as a valid test of directional pleiotropy, whereas the slope of the MR-Egger regression is the pleiotropy-adjusted causal effect estimate. We further used the weighted median method to diagnose and protect against invalid genetic instruments.

[b]The statistics could not calculated (and are denoted as not applicable [NA]) for pancreatic cancer and neuroblastoma, because less than three SNPs were available for these cancers.

[c]The odds ratios (ORs) represent increase/decrease of risk per 25 nmol/L increase in the natural scale of 25(OH)D.
