# Supplementary material for: Circulating vitamin D concentration and risk of seven cancers: Mendelian randomisation study
Source: BMJ. 2017 Oct 31;359:j4761. doi: 10.1136/bmj.j4761 (PMC5666592; doi:10.1136/bmj.j4761)
Supplement: Supplementary file 4 — Appendix 4: Further details of contributing consortiums, funding, and acknowledgments [file dimv039614.ww4.pdf]

Appendix 4: Further details of contributing consortiums, funding and acknowledgements [posted as supplied by authors]

The PRACTICAL Consortium (http://practical.ccge.medschl.cam.ac.uk/):

Rosalind Eeles, Professor[1,2], Doug Easton, Professor[3], Zsofia Kote-Jarai, Senior Researcher[1],Ali Amin Al Olama, Research Associate[3], Sara Benlloch, Research Associate[3], Kenneth Muir, Professor[4], Graham G. Giles, Professor[5,6], Fredrik Wiklund, Senior Lecturer[7], Henrik Gronberg, Professor[7], Christopher A. Haiman, Professor[8], Johanna Schleutker, Professor[9,10], Maren Weischer, Professor[11], Ruth C. Travis, Associate Professor[12], David Neal, Professor[13], Paul Pharoah, Professor[14], Kay-Tee Khaw, Professor[15], Janet L. Stanford, Professor[16,17], William J. Blot, Professor[18], Stephen Thibodeau, Professor[19], Christiane Maier, Professor[20], Adam S. Kibel, Professor[22,23], Cezary Cybulski, Professor[24], Lisa Cannon-Albright, Professor[25], Hermann Brenner, Professor[26,27,33], Jong Park, Professor[28], Radka Kaneva, Associate Professor[29], Jyotsna Batra, Senior Research Fellow[30], Manuel R. Teixeira, Professor[31], Hardev Pandha, Professor[32], Yong-Jie Lu, Reader[34]

[1] The Institute of Cancer Research, London, SM2 5NG, UK, [2] Royal Marsden NHS Foundation Trust, London, SW3 6JJ, UK, [3] Centre for Cancer Genetic Epidemiology, Department of Public Health and Primary Care, University of Cambridge, Strangeways Research Laboratory, Worts Causeway, Cambridge, UK, [4] University of Warwick, Coventry, UK, [5] Cancer Epidemiology Centre, Cancer Council Victoria, 615 St Kilda Road, Melbourne, Victoria, 3004, Australia, [6] Centre for Epidemiology and Biostatistics, Melbourne School of Population and Global Health, The University of Melbourne, Melbourne, Victoria, Australia, [7] Department of Medical Epidemiology and Biostatistics, Karolinska Institute, Stockholm, Sweden, [8] Department of Preventive Medicine, Keck School of Medicine, University of Southern California/Norris Comprehensive Cancer Center, Los Angeles, California, USA, [9] Department of Medical Biochemistry and Genetics, Institute of Biomedicine, Kiinamyllynkatu 10, FI-20014 University of Turku; and Tyks Microbiology and Genetics, Department of Medical Genetics, Turku University Hospital, [10] BioMediTech, 30014 University of Tampere, Tampere, Finland, [11] Department of Clinical Biochemistry, Herlev Hospital, Copenhagen University Hospital, HerlevRingvej 75, DK-2730 Herlev, Denmark, [12] Cancer Epidemiology, Nuffield Department of Population Health, University of Oxford, Oxford, OX3 7LF, UK, [13] Nuffield Department of Surgery, University of Oxford, Room 6603, Level 6, John Radcliffe Hospital, Headley Way, Headington, Oxford, OX3 9DU, UK and University of Cambridge, Department of Oncology, Box 279, Addenbrooke's Hospital, Hills Road Cambridge CB2 0QQ, UK, [14] Centre for Cancer Genetic Epidemiology, Department of Oncology, University of Cambridge, Strangeways Research Laboratory, Worts Causeway, Cambridge, UK, [15] Cambridge Institute of Public Health, University of Cambridge, Forvie Site, Robinson Way, Cambridge CB2 0SR, [16] Division of Public Health Sciences, Fred Hutchinson Cancer Research Center, Seattle, Washington, USA, [17] Department of Epidemiology, School of Public Health, University of Washington, Seattle, Washington, USA, [18] International Epidemiology Institute, 1455 Research Blvd., Suite 550, Rockville, MD 20850, [19] Mayo Clinic, Rochester, Minnesota, USA, [20]Institute of Human Genetics, University Hospital of Ulm, Ulm, Germany,[22] Brigham and Women's Hospital/Dana-Farber Cancer Institute,

45 Francis Street- ASB II-3, Boston, MA 02115, [23] Washington University, St Louis, Missouri, [24] International Hereditary Cancer Center, Department of Genetics and Pathology, Pomeranian Medical University, Szczecin, Poland, [25] Division of Genetic Epidemiology, Department of Medicine, University of Utah School of Medicine, [26] Division of Clinical Epidemiology and Aging Research, German Cancer Research Center (DKFZ), Heidelberg Germany, [27]Division of Preventive Oncology, German Cancer Research Center (DKFZ) and National Center for Tumor Diseases (NCT), Heidelberg, Germany, [28]Division of Cancer Prevention and Control, H. Lee Moffitt Cancer Center, 12902 Magnolia Dr., Tampa, Florida, USA, [29] Molecular Medicine Center and Department of Medical Chemistry and Biochemistry, Medical University - Sofia, 2 Zdrave St, 1431, Sofia, Bulgaria, [30] Australian Prostate Cancer Research Centre-Qld, Institute of Health and Biomedical Innovation and School of Biomedical Science, Queensland University of Technology, Brisbane, Australia, [31] Department of Genetics, Portuguese Oncology Institute, Porto, Portugal and Biomedical Sciences Institute (ICBAS), Porto University, Porto, Portugal, [32]The University of Surrey, Guildford, Surrey, GU2 7XH, UK, [33]German Cancer Consortium (DKTK), German Cancer Research Center (DKFZ), Heidelberg, Germany, [34]Centre for Molecular Oncology, Barts Cancer Institute, Queen Mary University of London, John Vane Science Centre, Charterhouse Square, London, EC1M 6BQ, UK

COGS & PRACTICAL acknowledgement and funding:

This study would not have been possible without the contributions of the following: Per Hall (COGS); Douglas F. Easton, Paul Pharoah, Kyriaki Michailidou, Manjeet K. Bolla, Qin Wang (BCAC), Andrew Berchuck (OCAC), Rosalind A. Eeles, Douglas F. Easton, Ali Amin Al Olama, Zsofia Kote-Jarai, Sara Benlloch (PRACTICAL), Georgia Chenevix-Trench, Antonis Antoniou, Lesley McGuffog, Fergus Couch and Ken Offit (CIMBA), Joe Dennis, Alison M. Dunning, Andrew Lee, and Ed Dicks, Craig Luccarini and the staff of the Centre for Genetic Epidemiology Laboratory, Javier Benitez, Anna Gonzalez-Neira and the staff of the CNIO genotyping unit, Jacques Simard and Daniel C. Tessier, Francois Bacot, Daniel Vincent, Sylvie LaBoissière and Frederic Robidoux and the staff of the McGill University and Génome Québec Innovation Centre, Stig E. Bojesen, Sune F. Nielsen, Borge G. Nordestgaard, and the staff of the Copenhagen DNA laboratory, and Julie M. Cunningham, Sharon A. Windebank, Christopher A. Hilker, Jeffrey Meyer and the staff of Mayo Clinic Genotyping Core Facility

Funding for the iCOGS infrastructure came from: the European Community's Seventh Framework Programme under grant agreement n° 223175 (HEALTH-F2-2009-223175) (COGS), Cancer Research UK (C1287/A10118, C1287/A 10710, C12292/A11174, C1281/A12014, C5047/A8384, C5047/A15007, C5047/A10692, C8197/A16565), the National Institutes of Health (CA128978) and Post-Cancer GWAS initiative (1U19 CA148537, 1U19 CA148065 and 1U19 CA148112 - the GAME-ON initiative), the Department of Defence (W81XWH-10-1-0341), the Canadian Institutes of Health Research (CIHR) for the CIHR Team in Familial Risks of Breast Cancer, Komen Foundation for the Cure, the Breast Cancer Research Foundation, and the Ovarian Cancer Research Fund.

Particularly the PRACTICAL consortium was supported by the Canadian Institutes of Health Research, European Commission's Seventh Framework Programme grant agreement n° 223175 (HEALTH-F2-2009-223175), Cancer Research UK Grants C5047/A7357, C1287/A10118, C5047/A3354, C5047/A10692, C16913/A6135, and The National Institute of Health (NIH) Cancer Post-Cancer GWAS initiative grant: No. 1 U19 CA 148537-01 (the GAME-ON initiative). PRACTICAL further acknowledges support from the NIHR to the Biomedical Research Centre at The Institute of Cancer Research and Royal Marsden NHS Foundation Trust.

GECCO Funding and Acknowledgments

Funding

GECCO: National Cancer Institute, National Institutes of Health, U.S. Department of Health and Human Services (U01 CA137088; R01 CA059045; R01 CA120582 ; U01 CA164930). Genotyping/Sequencing services was done by the Center for Inherited Disease Research (CIDR). CIDR is funded by the NIH to The Johns Hopkins University, contract number HHSN268201200008I.
ASTERISK: a Hospital Clinical Research Program (PHRC-BRD09/C) from the University Hospital Center of Nantes (CHU de Nantes) and supported by the Regional Council of Pays de la Loire, the Groupement des Entreprises Françaises dans la Luttecontre le Cancer (GEFLUC), the Association Anne de Bretagne Génétique and the Ligue Régionale Contre le Cancer (LRCC).
COLO2&3: National Institutes of Health (R01 CA60987).
DACHS: German Research Council (Deutsche Forschungsgemeinschaft, BR 1704/6-1, BR 1704/6-3, BR 1704/6-4 and CH 117/1-1), and the German Federal Ministry of Education and Research (01KH0404 and 01ER0814).
DALS: National Institutes of Health (R01 CA48998 to M. L. Slattery);
HPFS is supported by the National Institutes of Health (P01 CA055075, UM1 CA167552, R01 CA137178, R01 CA151993, R35 CA197735, K07 CA190673, and P50 CA127003), NHS by the National Institutes of Health (R01 CA137178, P01 CA087969, UM1 CA186107, R01 CA151993, R35 CA197735, K07 CA190673, and P50 CA127003,) and PHS by the National Institutes of Health (R01 CA042182).
MEC: National Institutes of Health (R37 CA54281, P01 CA033619, R01 CA63464and U01 CA164973).
OFCCR: National Institutes of Health, through funding allocated to the Ontario Registry for Studies of Familial Colorectal Cancer (U01 CA074783); see CCFR section above. Additional funding toward genetic analyses of OFCCR includes the Ontario Research Fund, the Canadian Institutes of Health Research, and the Ontario Institute for Cancer Research, through generous support from the Ontario Ministry of Research and Innovation.
PLCO: Intramural Research Program of the Division of Cancer Epidemiology and Genetics and supported by contracts from the Division of Cancer Prevention, National Cancer Institute, NIH, DHHS. Additionally, a subset of control samples were genotyped as part of the Cancer Genetic Markers of Susceptibility (CGEMS) Prostate Cancer GWAS (Yeager, M et al. Genome-wide association study of prostate cancer identifies a second risk locus at 8q24. Nat Genet 2007 May;39(5):645-9) and the Lung Cancer and Smoking study (Landi MT, et al. A genome-wide association study of lung cancer identifies a region of chromosome 5p15 associated with risk for

adenocarcinoma. Am J Hum Genet. 2009 Nov;85(5):679-91). The prostate study dataset was accessed with approval through the dbGaP online resource (http://cgems.cancer.gov/data/) accession number phs000207.v1.p1 and the lung datasets were accessed from the dbGaP website (http://www.ncbi.nlm.nih.gov/gap) through accession number phs000093.v2.p2. Funding for the Lung Cancer and Smoking study was provided by National Institutes of Health (NIH), Genes, Environment and Health Initiative (GEI) Z01 CP 010200, NIH U01 HG004446, and NIH GEI U01 HG 004438. For the lung study, the GENEVA Coordinating Center provided assistance with genotype cleaning and general study coordination, and the Johns Hopkins University Center for Inherited Disease Research conducted genotyping.
PMH: National Institutes of Health (R01 CA076366 to P.A. Newcomb).
VITAL: National Institutes of Health (K05 CA154337).
WHI: The WHI program is funded by the National Heart, Lung, and Blood Institute, National Institutes of Health, U.S. Department of Health and Human Services through contracts HHSN268201100046C, HHSN268201100001C, HHSN268201100002C, HHSN268201100003C, HHSN268201100004C, and HHSN271201100004C.

Acknowledgements

ASTERISK: We are very grateful to Dr. Bruno Buecher without whom this project would not have existed. We also thank all those who agreed to participate in this study, including the patients and the healthy control persons, as well as all the physicians, technicians and students.
DACHS: We thank all participants and cooperating clinicians, and Ute Handte-Daub, Utz Benscheid, Muhabbet Celik and Ursula Eilber for excellent technical assistance.
GECCO: The authors would like to thank all those at the GECCO Coordinating Center for helping bring together the data and people that made this project possible. The authors also acknowledge Deanna Stelling, Mark Thornquist, Greg Warnick, Carolyn Hutter, and team members at COMPASS (Comprehensive Center for the Advancement of Scientific Strategies) at the Fred Hutchinson Cancer Research Center for their work harmonizing the GECCO epidemiological data set. The authors acknowledge Dave Duggan and team members at TGEN (Translational Genomics Research Institute), the Broad Institute, and the Génome Québec Innovation Center for genotyping DNA samples of cases and controls, and for scientific input for GECCO.
HPFS, NHS and PHS: We would like to acknowledge Patrice Soule and Hardeep Ranu of the Dana Farber Harvard Cancer Center High-Throughput Polymorphism Core who assisted in the genotyping for NHS, HPFS, and PHS under the supervision of Dr. Immaculata Devivo and Dr. David Hunter, Qin (Carolyn) Guo and Lixue Zhu who assisted in programming for NHS and HPFS, and Haiyan Zhang who assisted in programming for the PHS. We would like to thank the participants and staff of the Nurses' Health Study and the Health Professionals Follow-Up Study, for their valuable contributions as well as the following state cancer registries for their help: AL, AZ, AR, CA, CO, CT, DE, FL, GA, ID, IL, IN, IA, KY, LA, ME, MD, MA, MI, NE, NH, NJ, NY, NC, ND, OH, OK, OR, PA, RI, SC, TN, TX, VA, WA, WY. The authors assume full responsibility for analyses and interpretation of these data.
PLCO: The authors thank Drs. Christine Berg and Philip Prorok, Division of Cancer Prevention, National Cancer Institute, the Screening Center investigators and staff or

the Prostate, Lung, Colorectal, and Ovarian (PLCO) Cancer Screening Trial, Mr. Tom Riley and staff, Information Management Services, Inc., Ms. Barbara O'Brien and staff, Westat, Inc., and Drs. Bill Kopp and staff, SAIC-Frederick. Most importantly, we acknowledge the study participants for their contributions to making this study possible. The statements contained herein are solely those of the authors and do not represent or imply concurrence or endorsement by NCI.
PMH: The authors would like to thank the study participants and staff of the Hormones and Colon Cancer study.
WHI: The authors thank the WHI investigators and staff for their dedication, and the study participants for making the program possible. A full listing of WHI investigators can be found at: http://www.whi.org/researchers/Documents%20%20Write%20a%20Paper/WHI%20Investigator%20Short%20List.pdf

CORECT acknowledgement and funding:

Colon Cancer Family Registry funding:

This work was supported by grant UM1 CA167551 from the National Cancer Institute and through cooperative agreements with the following Colon Cancer Family Registry centres: Australasian Colorectal Cancer Family Registry (U01 CA074778 and U01/U24 CA097735); Mayo Clinic Cooperative Family Registry for Colon Cancer Studies (U01/U24 CA074800); Ontario Familial Colorectal Cancer Registry (U01/U24 CA074783); and Seattle Colorectal Cancer Family Registry (U01/U24 CA074794). Seattle CCFR research was also supported by the Cancer Surveillance System of the Fred Hutchinson Cancer Research Center, which was funded by Control Nos. N01-CN-67009 (1996-2003) and N01-PC-35142 (2003-2010) and Contract No. HHSN2612013000121 (2010-2017) from the Surveillance, Epidemiology and End Results (SEER) Program of the National Cancer Institute with additional support from the Fred Hutchinson Cancer Research Center. MEC was funded by U01 CA164973.

MCCS cohort recruitment was funded by VicHealth and Cancer Council Victoria. The MCCS was further supported by Australian NHMRC grants 209057, 251553 and 504711 and by infrastructure provided by Cancer Council Victoria. Cases and their vital status were ascertained through the Victorian Cancer Registry (VCR) and the Australian Institute of Health and Welfare (AIHW), including the National Death Index and the Australian Cancer Database.

The coordination of EPIC is financially supported by the European Commission (DG-SANCO) and the International Agency for Research on Cancer. The national cohorts are supported by Danish Cancer Society (Denmark); German Cancer Aid, German Cancer Research Center (DKFZ), Federal Ministry of Education and Research (BMBF), Deutsche Krebshilfe, Deutsches Krebsforschungszentrum and Federal Ministry of Education and Research (Germany); the Hellenic Health Foundation (Greece); Associazione Italiana per la Ricercasul Cancro-AIRC-Italy and National Research Council (Italy); Dutch Ministry of Public Health, Welfare and Sports (VWS), Netherlands Cancer Registry (NKR), LK Research Funds, Dutch Prevention Funds, Dutch ZON (Zorg Onderzoek Nederland), World Cancer Research Fund

(WCRF), Statistics Netherlands (The Netherlands); Health Research Fund (FIS) PI13/00061 (EPIC-Granada) and, PI13/01162 (EPIC-Murcia), Regional Governments of Andalucía, Asturias, Basque Country, Murcia and Navarra, ISCIII Health Research Funds RD12/0036/0018 (cofounded by FEDER funds/European Regional Development Fund ERDF) (Spain); Swedish Cancer Society, Swedish Research Council and County Councils of Skåne and Västerbotten (Sweden); Cancer Research UK (14136 to EPIC-Norfolk; C570/A16491 and C8221/A19170 for EPIC-Oxford), Medical Research Council (1000143 to EPIC-Norfolk, MR/M012190/1 to EPIC-Oxford) (UK). For information on how to submit an application for gaining access to EPIC data and/or biospecimens, please follow the instructions at http://epic.iarc.fr/access/index.php.

DRIVE Funding:

BCFR: NIH R01 CA122171 (Ahsan)
